# Rising burden of subarachnoid hemorrhage linked to high systolic blood pressure among young and middle-aged populations: temporal trends and global implication

**DOI:** 10.3389/fnhum.2025.1700918

**Published:** 2025-10-30

**Authors:** Pengfei Chen, Junlin Chen, Jiazuo Liu

**Affiliations:** ^1^Department of Critical Care Medicine, Dazhou Central Hospital, Dazhou, Sichuan, China; ^2^Department of Interventional Radiology, Dazhou Central Hospital, Dazhou, Sichuan, China

**Keywords:** subarachnoid hemorrhage, high systolic blood pressure, young adults, global burden, temporal trends

## Abstract

**Background:**

Subarachnoid hemorrhage (SAH) is a devastating cerebrovascular event that leads to high mortality and long-term disability, particularly among adults aged 25–49 years. Elevated systolic blood pressure (SBP) is the leading modifiable risk factor, yet its global burden in this age group has not been systematically assessed.

**Methods:**

We analyzed population-level epidemiological data across 204 countries and territories from 1990 to 2021 to estimate SAH mortality and disability-adjusted life-years (DALYs) attributable to high SBP. Temporal trends were evaluated using estimated annual percentage change (EAPC), and future trajectories to 2050 were projected with autoregressive integrated moving average (ARIMA) models.

**Results:**

Globally, SAH caused 24,908 deaths and 1,373,366 DALYs in 2021 due to high SBP in adults aged 25–49 years. Males had higher rates than females (mortality: 0.78 vs. 0.48 per 100,000; DALYs: 42.4 vs. 27.0 per 100,000), though females experienced steeper declines (EAPC_mortality: −1.78%). The highest burden was in the 45–49 age group (deaths: 9,768; DALYs: 474,092). From 1990 to 2021, high-SDI regions achieved the greatest reductions in mortality (−50.3%) and DALYs (−46.8%), while low-SDI regions saw increases in deaths (+109.5%) and DALYs (+114.7%). Nationally, Zimbabwe and Guatemala showed the sharpest increases in mortality (EAPC: +4.71% and +3.76%), while Sweden and Swiss Confederation achieved the greatest declines (EAPC: −5.5% and −4.9%). Forecasts suggest continued global declines by 2050, but widening disparities by sex, age, and socio-demographic status.

**Conclusion:**

Although the global burden of SAH attributable to high SBP is decreasing, young and middle-aged adults-especially males and those in low-SDI regions-continue to face substantial risks. Targeted hypertension control and region-specific prevention strategies are urgently needed.

## Introduction

Subarachnoid hemorrhage (SAH) is a serious and often fatal type of stroke caused by bleeding into the subarachnoid space (Rautalin et al., 2024). Although it represents a relatively small proportion of all stroke cases globally, SAH leads to considerable mortality and disability, particularly among individuals of working age (Haripottawekul et al., 2025; Wyckoff and Hsiang-Yi Chou, 2025). The high case fatality rate and long-term neurological sequelae in survivors make SAH a major contributor to premature death and loss of productive life-years, imposing a significant burden on families, health systems, and societies, especially in low- and middle-income countries (Poliseli et al., 2024).

Among the risk factors for SAH, elevated systolic blood pressure (SBP) is the most important modifiable cause (Eagles et al., 2025). Persistent high SBP increases the risk of aneurysm formation and rupture by promoting structural changes in cerebral vessels (Lin et al., 2023). Despite improvements in hypertension awareness and management in many parts of the world, the prevalence of uncontrolled high SBP remains substantial (GBD 2021 Global Subarachnoid Hemorrhage Risk Factors Collaborators, Rautalin et al., 2025). This is particularly concerning among young and middle-aged adults, who are less likely to be screened or treated for hypertension compared to older adults (Betteridge et al., 2024). The long-term vascular damage associated with early-onset high SBP not only increases the lifetime risk of SAH, but also contributes to other forms of premature cardiovascular disease (Frontera et al., 2010).

In this study, we aimed to describe the global and regional burden of SAH attributable to high SBP among young and middle-aged adults from 1990 to 2021, using data from the Global Burden of Disease (GBD) Study (GBD 2021 Causes of Death Collaborators, 2024). We also sought to examine temporal trends in mortality, incidence, and disability-adjusted life-years (DALYs) related to high SBP in this population. Our findings are intended to provide evidence to support targeted public health interventions to reduce preventable SAH deaths and disability in working-age adults.

## Materials and methods

### Data sources and study population

This study used data from the GBD Study 2021, coordinated by the Institute for Health Metrics and Evaluation (IHME) (GBD 2021 Causes of Death Collaborators, 2024; GBD 2021 Diseases and Injuries Collaborators, 2024). GBD provides standardized estimates of disease burden attributable to various causes and risk factors across 204 countries and territories, 21 geographical regions, and five Socio-demographic Index (SDI) levels, covering the years 1990–2021 (Zhang et al., 2023). Data were downloaded from the Global Health Data Exchange^1^ on 25 May 2025. Estimates were based on data from vital registration systems, verbal autopsy, hospital records, claims data, surveys, and published literature, modeled using the DisMod-MR 2.1 Bayesian meta-regression framework (Fan et al., 2022). This analysis focused on adults aged 25–49 years, stratified into 5-year age groups (25–29, 30–34, 35–39, 40–44, 45–49 years) and by sex. Data were analyzed at global, SDI quintile, regional (21 regions), and national (204 countries and territories) levels.

### Definition of subarachnoid hemorrhage and high systolic blood pressure

Subarachnoid hemorrhage was defined as bleeding into the subarachnoid space, typically resulting from rupture of an intracranial aneurysm or vascular malformation (Lee et al., 2025). SAH was classified using the following International Classification of Diseases (ICD) codes: ICD-10 I60-I60.9 (non-traumatic SAH), I67.0-I67.1 (cerebral artery dissection and arteriovenous malformations); ICD-9 430-430.9 (non-traumatic SAH), 431.0 (intracranial hemorrhage involving subarachnoid space), and 437.3 (arteriovenous malformations) (Lv et al., 2024). High SBP was defined as SBP ≥ 115 mmHg, with a theoretical minimum risk exposure level (TMREL) of 110–115 mmHg in line with GBD comparative risk assessment standards. The burden attributable to high SBP was calculated as the proportion of SAH burden linked to SBP levels above the TMREL (GBD 2021 Global Subarachnoid Hemorrhage Risk Factors Collaborators, Rautalin et al., 2025).

### Statistical analysis

Temporal trends from 1990 to 2021 were assessed using estimated annual percentage change (EAPC). Log-linear regression models were fitted to the natural logarithm of crude incidence, mortality, and DALY rates: ln(rate) = α + β × year + γ; EAPC was calculated as: EAPC = 100 × (e^β^−1) (Lv et al., 2024).

A trend was deemed significant if both the EAPC estimate and its 95% confidence interval (CI) were either above or below zero. In addition, autoregressive integrated moving average (ARIMA) models were used to forecast incidence, mortality, and DALYs up to 2050. Model parameters (p, d, q) were selected based on autocorrelation and partial autocorrelation plots and AIC minimization, with model adequacy confirmed by residual diagnostics (Zhang Y. F. et al., 2025).

## Results

### Global burden and temporal trends of SAH attributable to high SBP

**FIGURE 1:**
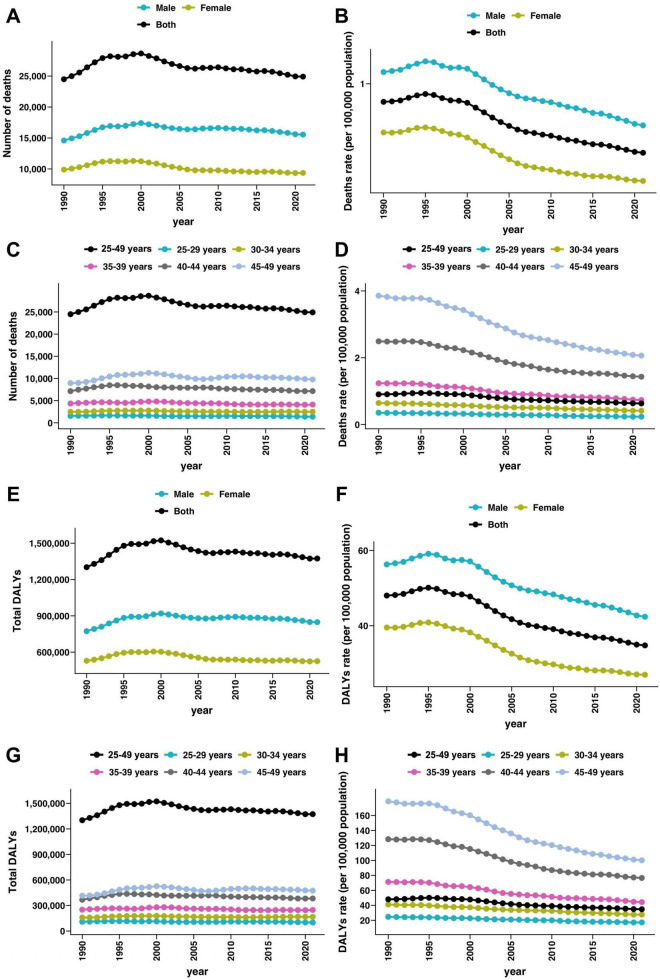
Global trends in subarachnoid hemorrhage deaths and disability-adjusted life years (DALYs) attributable to high systolic blood pressure among young and middle-aged adults from 1990 to 2021. **(A,B)** Number of deaths and sex-specific death rates (per 100,000 population) due to subarachnoid hemorrhage attributable to high systolic blood pressure over time. **(C,D)** Number of deaths and age-specific death rates (per 100,000 population) due to subarachnoid hemorrhage attributable to high systolic blood pressure by age group (25–49 years overall and 5-year subgroups) over time. **(E,F)** Number of DALYs and sex-specific DALYs rate (per 100,000 population) due to subarachnoid hemorrhage attributable to high systolic blood pressure over time. **(G,H)** Number of DALYs and sex-specific DALYs rate (per 100,000 population) due to subarachnoid hemorrhage attributable to high systolic blood pressure by age group (25–49 years overall and 5-year subgroups) over time.

In 2021, there were an estimated 24,908 [95% uncertainty interval (UI) 16,515 to 34,310] deaths globally from SAH attributable to high SBP among adults aged 25–49 years, corresponding to a crude mortality rate of 0.63 (95% UI 0.42 to 0.87) per 100,000 population. This represented a slight increase of 1.72% (95% UI −13.32 to 19.63) in the absolute number of deaths compared with 1990 (Figure 1A and Table 1). The global mortality rate declined more markedly over this period, with an EAPC of −1.44% (95% CI −1.56 to −1.31). Male adults consistently experienced a higher mortality burden compared with females. In 2021, the crude mortality rate among males was 0.78 (95% UI 0.50 to 1.15) per 100,000 population, compared to 0.48 (95% UI 0.32 to 0.66) in females (Figure 1B and Table 1). Although the number of SAH deaths attributable to high SBP in males increased modestly by 6.54% (95% UI −12.15 to 34.36) from 1990 to 2021, the mortality rate declined significantly, with an EAPC of −1.22% (95% CI −1.34 to −1.11). In females, both the number of deaths and the mortality rate decreased, with an EAPC of −1.78% (95% CI −1.93 to −1.62) (Table 1).

**TABLE 1:** Global and regional deaths and trends of subarachnoid hemorrhage attributable to high systolic blood pressure among 25–49 aged from 1990 to 2021.

Feature	Cases_1990	Rates_1990	Cases_2021	Rates_2021	Cases_change	EAPC_CI
Global	24,488 (15,743 to 34,197)	0.9 (0.58 to 1.26)	24,908 (16,515 to 34,310)	0.63 (0.42 to 0.87)	1.72 (−13.32 to 19.63)	−1.44 (−1.56 to −1.31)
**Sex group**						
Female	9,892 (6,117 to 14,011)	0.74 (0.46 to 1.05)	9,357 (6,200 to 12,783)	0.48 (0.32 to 0.66)	−5.41 (−22.92 to 24.6)	−1.78 (−1.93 to −1.62)
Male	14,595 (8,790 to 21,900)	1.06 (0.64 to 1.59)	15,550 (10,025 to 23,060)	0.78 (0.5 to 1.15)	6.54 (−12.15 to 34.36)	−1.22 (−1.34 to −1.11)
**Age group**						
25–29 years	1,559 (894 to 2,518)	0.35 (0.2 to 0.57)	1,377 (806 to 2,166)	0.23 (0.14 to 0.37)	−11.71 (−30.59 to 14.72)	−1.43 (−1.49 to −1.38)
30–34 years	2,484 (1,475 to 3,675)	0.64 (0.38 to 0.95)	2,498 (1,487 to 3,699)	0.41 (0.25 to 0.61)	0.57 (−19.05 to 27.75)	−1.5 (−1.55 to −1.46)
35–39 years	4,354 (2,618 to 6,422)	1.24 (0.74 to 1.82)	4,110 (2,617 to 5,752)	0.73 (0.47 to 1.03)	−5.61 (−25.33 to 21.24)	−1.84 (−1.93 to −1.74)
40–44 years	7,137 (4,610 to 10,330)	2.49 (1.61 to 3.61)	7,155 (4,676 to 9,931)	1.43 (0.93 to 1.99)	0.25 (−18.73 to 23.88)	−2.11 (−2.23 to −1.98)
45–49 years	8,953 (5,691 to 12,579)	3.86 (2.45 to 5.42)	9,768 (6,661 to 13,142)	2.06 (1.41 to 2.78)	9.11 (−10.35 to 35.09)	−2.3 (−2.41 to −2.19)
**SDI regions**						
High SDI	4,879 (3,400 to 6,226)	1.06 (0.74 to 1.35)	2,425 (1,597 to 3,136)	0.48 (0.32 to 0.62)	−50.3 (−55.08 to −45.85)	−2.92 (−3.11 to −2.73)
High-middle SDI	5,143 (3,278 to 7,027)	0.91 (0.58 to 1.24)	3,887 (2,704 to 4,969)	0.62 (0.43 to 0.79)	−24.42 (−39.17 to −4.96)	−1.88 (−2.18 to −1.58)
Middle SDI	8,659 (5,128 to 12,685)	0.95 (0.56 to 1.39)	8,619 (5,831 to 11,316)	0.69 (0.46 to 0.9)	−0.45 (−23.35 to 32.89)	−1.31 (−1.46 to −1.17)
Low-middle SDI	4,589 (2,684 to 7,069)	0.83 (0.49 to 1.28)	7,456 (4,659 to 11,168)	0.73 (0.46 to 1.1)	62.48 (36.94 to 97.55)	−0.34 (−0.42 to −0.27)
Low SDI	1,189 (542 to 2,382)	0.54 (0.25 to 1.08)	2492 (1116 to 5474)	0.46 (0.21 to 1.01)	109.54 (65.93 to 161.79)	−0.57 (−0.62 to −0.52)
**Geographical regions**						
Andean Latin America	113 (54 to 183)	0.61 (0.29 to 0.98)	277 (169 to 409)	0.79 (0.48 to 1.17)	144.97 (62.87 to 291.64)	1.35 (1.04 to 1.66)
Australasia	81 (55 to 107)	0.75 (0.51 to 1)	58 (36 to 80)	0.4 (0.25 to 0.55)	−28.64 (−42.47 to −13.93)	−2.38 (−2.62 to −2.15)
Caribbean	186 (114 to 267)	1.02 (0.62 to 1.46)	306 (180 to 453)	1.28 (0.75 to 1.89)	64.78 (20.63 to 133.78)	0.93 (0.81 to 1.05)
Central Asia	215 (148 to 276)	0.65 (0.44 to 0.83)	357 (257 to 462)	0.73 (0.53 to 0.95)	65.78 (33.09 to 102.98)	0.17 (−0.13 to 0.47)
Central Europe	956 (691 to 1,185)	1.54 (1.11 to 1.91)	457 (331 to 567)	0.87 (0.63 to 1.08)	−52.21 (−57.65 to −45.75)	−2.09 (−2.35 to −1.84)
Central Latin America	522 (345 to 705)	0.64 (0.42 to 0.86)	997 (660 to 1346)	0.75 (0.5 to 1.01)	90.97 (57.56 to 137.25)	0.47 (0.36 to 0.59)
Central Sub-Saharan Africa	95 (38 to 213)	0.39 (0.16 to 0.87)	225 (75 to 675)	0.34 (0.11 to 1.03)	136.77 (39.67 to 307.25)	−0.52 (−0.67 to −0.37)
East Asia	6,407 (2,863 to 10,481)	0.93 (0.42 to 1.52)	3,287 (1,859 to 4,922)	0.48 (0.27 to 0.71)	−48.69 (−68.31 to −8.7)	−2.81 (−3.27 to −2.36)
Eastern Europe	1,308 (951 to 1,604)	1.19 (0.86 to 1.45)	1,493 (1,051 to 1,849)	1.55 (1.09 to 1.92)	14.16 (2.42 to 26.94)	−0.1 (−0.71 to 0.51)
Eastern Sub-Saharan Africa	341 (103 to 933)	0.41 (0.12 to 1.12)	799 (241 to 2,377)	0.38 (0.12 to 1.14)	134.2 (81.7 to 199.59)	−0.29 (−0.34 to −0.23)
High-income Asia Pacific	1,698 (1,180 to 2,176)	1.83 (1.27 to 2.34)	674 (444 to 903)	0.86 (0.57 to 1.15)	−60.31 (−68.75 to −51.38)	−2.85 (−3.13 to −2.57)
High-income North America	1,018 (654 to 1,369)	0.68 (0.44 to 0.92)	751 (459 to 1,013)	0.45 (0.27 to 0.6)	−26.25 (−38.41 to −11.34)	−1.81 (−2.14 to −1.49)
North Africa and Middle East	960 (563 to 1,450)	0.6 (0.35 to 0.9)	1,170 (714 to 1,695)	0.35 (0.21 to 0.51)	21.88 (−20.27 to 62.02)	−1.92 (−2.06 to −1.79)
Oceania	29 (15 to 49)	0.9 (0.47 to 1.54)	77 (40 to 134)	1.1 (0.57 to 1.9)	169.73 (79.95 to 298.36)	0.72 (0.52 to 0.92)
South Asia	4,268 (2,300 to 7,033)	0.81 (0.43 to 1.33)	6,781 (4,057 to 10,468)	0.67 (0.4 to 1.04)	58.89 (28.41 to 106.08)	−0.57 (−0.65 to −0.49)
Southeast Asia	2,308 (1,474 to 3,487)	0.98 (0.62 to 1.47)	3,786 (2,523 to 5,257)	1.02 (0.68 to 1.42)	64.05 (28.08 to 111.93)	0.42 (0.23 to 0.61)
Southern Latin America	335 (204 to 487)	1.37 (0.83 to 1.99)	253 (163 to 339)	0.73 (0.47 to 0.98)	−24.48 (−42.72 to −0.4)	−1.88 (−2 to −1.77)
Southern Sub-Saharan Africa	80 (53 to 115)	0.31 (0.21 to 0.44)	164 (108 to 244)	0.38 (0.25 to 0.56)	104.74 (57.78 to 159.75)	0.92 (0.49 to 1.35)
Tropical Latin America	1,489 (998 to 1,945)	1.9 (1.27 to 2.48)	1,520 (1,059 to 1,969)	1.27 (0.88 to 1.64)	2.11 (−8.13 to 13.25)	−1.68 (−1.9 to −1.46)
Western Europe	1,769 (1,276 to 2,202)	0.91 (0.66 to 1.14)	720 (496 to 912)	0.38 (0.26 to 0.48)	−59.32 (−62.98 to −55.63)	−2.94 (−3.18 to −2.71)
Western Sub-Saharan Africa	308 (112 to 823)	0.36 (0.13 to 0.96)	755 (305 to 1,949)	0.33 (0.13 to 0.85)	145.13 (78.91 to 231.99)	−0.15 (−0.33 to 0.03)

The global burden of disability, measured by DALYs, followed a similar trend. In 2021, SAH attributable to high SBP accounted for 1,373,366 (95% UI 910,290 to 1,871,996) DALYs globally among adults aged 25–49 years, representing a crude rate of 34.78 (95% UI 23.05 to 47.41) per 100,000 population (Figures 1E, F and Supplementary Table 1). This reflects a slight decrease of 5.5% (95% UI −17.94 to 27.97) in DALYs compared with 1990, when the DALYs were estimated at 1,301,770 (95% UI 844,050 to 1,811,022). The DALY rate declined significantly over the study period, with an EAPC of −1.31% (95% CI −1.42 to −1.19). Again, the burden was higher in males than females. In 2021, the DALY rate in males was 42.4 (95% UI 28.36 to 61.22) per 100,000, compared to 26.97 (95% UI 18.16 to 36.75) in females (Figures 1E, F and Supplementary Table 1). The number of DALYs among males increased by 9.67% (95% UI −7.94 to 35.54) from 1990 to 2021, yet the DALY rate declined with an EAPC of −1.12% (95% CI −1.23 to −1.02). In contrast, females showed a reduction in both DALY number and rate, with an EAPC of −1.59% (95% CI −1.73 to −1.45) (Supplementary Table 1). The temporal trends illustrated a steady decline in both mortality and DALY rates across sexes from 1990 to 2021. The declines were more pronounced among females, while males continued to experience a relatively higher burden throughout the period.

### Age-specific patterns and trends

From 1990 to 2021, the global burden of SAH due to high SBP declined across all adult age groups (25–49 years). The 45–49 age group had the highest burden in 2021, with 4,163 deaths (crude mortality rate: 1.77 per 100,000), reflecting a 3.1% increase in deaths but a significant decline in mortality rate (EAPC −2.65%) (Figures 1C, D and Table 1). This group also had the greatest DALY burden (206,858 DALYs, rate: 87.78 per 100,000), with an 8.93% increase in DALYs but a notable rate reduction (EAPC −2.45%) (Figures 1G, H and Supplementary Table 1). The 40–44 age group had 2,698 deaths and 148,972 DALYs in 2021, with mortality and DALY rates declining at EAPCs of −2.48% and −2.28%, respectively (Table 1 and Supplementary Table 1). Among younger adults, the burden was lower but also steadily declined. The 25–29 age group saw a 22.98% decrease in deaths, with mortality and DALY rates dropping significantly (EAPC −1.95% and −1.66%) (Figures 1C, D, G, H). Similarly, the 30–34 and 35–39 age groups experienced declines in mortality and DALY rates at EAPCs of −2.12% to −2.26% (Table 1 and Supplementary Table 1). Although absolute numbers of deaths and DALYs remained highest in older age groups, all age bands saw significant reductions in crude rates, with steeper declines in older adults.

### Regional and SDI-level variations

**FIGURE 2:**
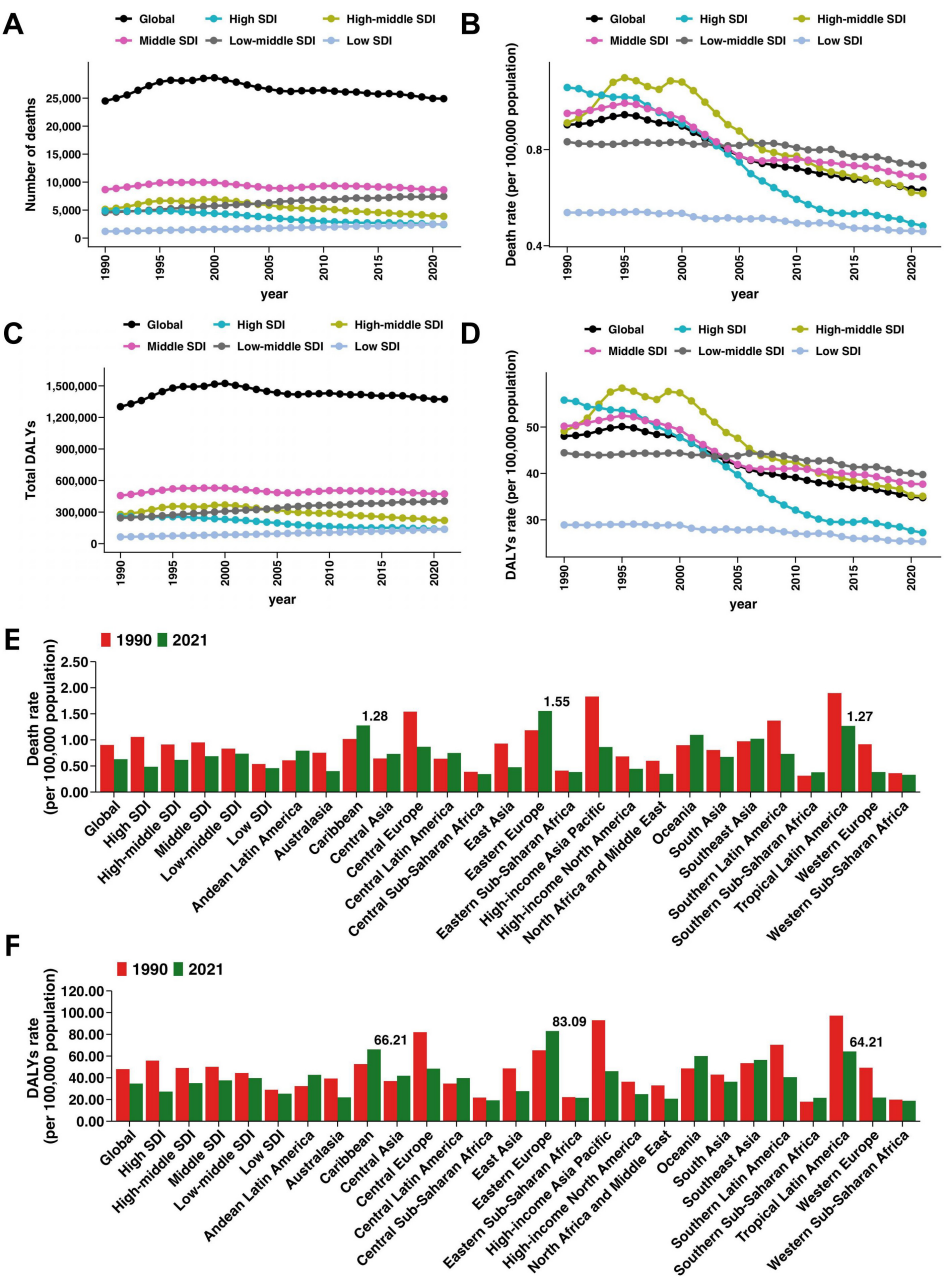
Regional patterns of subarachnoid hemorrhage mortality and disability-adjusted life years (DALYs) attributable to high systolic blood pressure among young and middle-aged adults. **(A)** Number of deaths by Socio-demographic Index (SDI) region and globally. **(B)** Death rates (per 100,000 population) over time by SDI region and globally. **(C)** Total DALYs by SDI region and globally. **(D)** DALY rates (per 100,000 population) over time by SDI region and globally. **(E)** Death rates in 1990 and 2021 at the global, SDI, and regional levels. **(F)** DALY rates in 1990 and 2021 at the global, SDI, and regional levels.

From 1990 to 2021, the mortality and DALY burden of SAH attributable to high SBP showed substantial variation across SDI and geographical regions. High SDI regions achieved the greatest reductions, with deaths decreasing by 50.3% (95% UI −55.08 to −45.85) and DALYs by 46.83% (95% UI −51.74 to −42.42). Mortality and DALY rates in these regions declined from 1.06 to 0.48 and from 55.8 to 27.22 per 100,000, respectively, with EAPCs of −2.92% and −2.69% (Figures 2A–D and Tables 1, 2). High-middle SDI regions also showed marked reductions in deaths (−24.42%, 95% UI −39.17 to −4.96) and DALYs (−20.32, 95% UI −35.04 to −1.13), with EAPCs of −1.88% and −1.67%. Middle SDI regions maintained relatively stable mortality rates (−0.45%, 95% UI −23.35 to 32.89) and DALYs (3.5%, 95% UI −19.32 to 36.39). In contrast, low-middle and low SDI regions experienced substantial increases in both deaths (62.48% and 109.54%) and DALYs (64.92% and 114.77%), though mortality and DALY rates declined slightly (EAPCs −0.34% to −0.57% for mortality, −0.31% to −0.48% for DALYs) (Figures 2A–D and Tables 1, 2).

**TABLE 2:** Global and regional disability-adjusted life-years (DALYs) and trends of subarachnoid hemorrhage attributable to high systolic blood pressure among 25–49 aged from 1990 to 2021.

Feature	Cases_1990	Rates_1990	Cases_2021	Rates_2021	Cases_change	EAPC_CI
Global	1301,770 (844,050 to 1,811,022)	48.03 (31.14 to 66.82)	1,373,366 (910,290 to 1,871,996)	34.78 (23.05 to 47.41)	5.5 (−9.75 to 22.3)	−1.31 (−1.42 to −1.19)
**Sex group**						
Female	528,734 (329,828 to 738,033)	39.54 (24.66 to 55.19)	525,577 (353,937 to 716,231)	26.97 (18.16 to 36.75)	−0.6 (−17.94 to 27.97)	−1.59 (−1.73 to −1.45)
Male	773,036 (470,516 to 1,150,603)	56.3 (34.27 to 83.8)	847,789 (567,158 to 1,224,213)	42.4 (28.36 to 61.22)	9.67 (−7.94 to 35.54)	−1.12 (−1.23 to −1.02)
**Age group**						
25–29 years	109,155 (62,420 to 174,314)	24.66 (14.1 to 39.38)	100,874 (60,450 to 157,823)	17.15 (10.27 to 26.82)	−7.59 (−27.08 to 18.43)	−1.27 (−1.32 to −1.23)
30–34 years	158,747 (94,880 to 234,989)	41.19 (24.62 to 60.97)	167,210 (103,985 to 245,806)	27.66 (17.2 to 40.66)	5.33 (−14.82 to 31.54)	−1.35 (−1.39 to −1.32)
35–39 years	250,989 (152,784 to 367,996)	71.25 (43.37 to 104.47)	248,286 (160,751 to 346,138)	44.27 (28.66 to 61.71)	−1.08 (−21.18 to 25.56)	−1.69 (−1.77 to −1.6)
40–44 years	367,606 (239,871 to 524,965)	128.32 (83.73 to 183.25)	382,904 (250,805 to 529,126)	76.54 (50.14 to 105.77)	4.16 (−15.03 to 27.66)	−1.97 (−2.09 to −1.85)
45–49 years	415,274 (269,892 to 574,802)	178.85 (116.23 to 247.55)	474,092 (327,657 to 634,374)	100.12 (69.2 to 133.97)	14.16 (−5.68 to 40.14)	−2.15 (−2.26 to −2.04)
**SDI regions**						
High SDI	257,149 (179,546 to 332,304)	55.8 (38.96 to 72.11)	136,726 (90,386 to 179,979)	27.22 (18 to 35.84)	−46.83 (−51.74 to −42.42)	−2.69 (−2.87 to −2.51)
High-middle SDI	277,247 (177,049 to 373,706)	49.12 (31.37 to 66.21)	220,909 (153,390 to 282,799)	35.09 (24.36 to 44.92)	−20.32 (−35.04 to −1.13)	−1.67 (−1.93 to −1.4)
Middle SDI	456,959 (268,870 to 667,106)	50.18 (29.53 to 73.26)	472,948 (320,255 to 623,500)	37.68 (25.52 to 49.68)	3.5 (−19.32 to 36.39)	−1.18 (−1.32 to −1.05)
Low-middle SDI	244,975 (147,577 to 375,134)	44.45 (26.78 to 68.07)	404,020 (256,757 to 602,961)	39.76 (25.27 to 59.33)	64.92 (40.24 to 97.89)	−0.31 (−0.38 to −0.24)
Low SDI	63,896 (31,487 to 122,936)	28.91 (14.24 to 55.62)	137,230 (66,770 to 288,291)	25.3 (12.31 to 53.15)	114.77 (73.94 to 163.35)	−0.48 (−0.53 to −0.43)
**Geographical regions**						
Andean Latin America	6,012 (2,832 to 9,694)	32.26 (15.2 to 52.02)	14,952 (9,084 to 21,790)	42.75 (25.97 to 62.3)	148.71 (67.8 to 295.25)	1.43 (1.11 to 1.75)
Australasia	4,239 (2,860 to 5,613)	39.28 (26.5 to 52.01)	3,179 (1,944 to 4,386)	22.02 (13.46 to 30.37)	−25 (−38.98 to −11.23)	−2.24 (−2.47 to −2.02)
Caribbean	9,603 (5,849 to 13,783)	52.57 (32.02 to 75.45)	15,854 (9,478 to 23,271)	66.21 (39.58 to 97.19)	65.09 (22.15 to 132.11)	0.94 (0.81 to 1.06)
Central Asia	12,321 (8,446 to 15,652)	36.95 (25.33 to 46.94)	20,376 (14,634 to 26,339)	41.79 (30.01 to 54.02)	65.37 (36.17 to 100.75)	0.16 (−0.08 to 0.4)
Central Europe	50,907 (36,665 to 64,017)	81.98 (59.05 to 103.09)	25,510 (18,482 to 31,664)	48.42 (35.08 to 60.09)	−49.89 (−55.2 to −43.83)	−1.89 (−2.09 to −1.69)
Central Latin America	28,252 (18,262 to 38,165)	34.61 (22.37 to 46.76)	52,879 (34,787 to 71,251)	39.72 (26.13 to 53.52)	87.17 (54.83 to 132.43)	0.42 (0.29 to 0.54)
Central Sub-Saharan Africa	5,310 (2,380 to 11,203)	21.75 (9.75 to 45.88)	12,582 (4,945 to 34,615)	19.3 (7.58 to 53.09)	136.96 (53.42 to 284.14)	−0.54 (−0.68 to −0.39)
East Asia	334,938 (157,284 to 539,980)	48.62 (22.83 to 78.39)	190,938 (112,361 to 276,511)	27.73 (16.32 to 40.16)	−42.99 (−64.43 to −3.83)	−2.47 (−2.87 to −2.06)
Eastern Europe	72,060 (52,546 to 88,658)	65.34 (47.64 to 80.39)	79,953 (56,724 to 98,777)	83.09 (58.95 to 102.65)	10.95 (−0.21 to 22.48)	−0.08 (−0.61 to 0.46)
Eastern Sub-Saharan Africa	18,610 (6,439 to 47,708)	22.31 (7.72 to 57.19)	45,075 (16,155 to 125,154)	21.53 (7.71 to 59.77)	142.21 (94.43 to 202.85)	−0.15 (−0.21 to −0.1)
High-income Asia Pacific	86,230 (60,504 to 110,274)	92.9 (65.18 to 118.8)	36,052 (23,784 to 47,888)	46.09 (30.41 to 61.22)	−58.19 (−66.74 to −49.12)	−2.67 (−2.95 to −2.39)
High-income North America	54,214 (35,052 to 73,781)	36.38 (23.52 to 49.51)	42,004 (25,812 to 57,826)	24.9 (15.3 to 34.28)	−22.52 (−35.45 to −7)	−1.62 (−1.93 to −1.3)
North Africa and Middle East	52,908 (32,295 to 77,935)	33.01 (20.15 to 48.63)	69,232 (44,110 to 97,699)	20.71 (13.19 to 29.22)	30.86 (−9.19 to 68.43)	−1.67 (−1.79 to −1.55)
Oceania	1,554 (825 to 2,629)	48.65 (25.81 to 82.29)	4,246 (2,268 to 7,129)	60.02 (32.05 to 100.77)	173.17 (91.3 to 293.52)	0.75 (0.54 to 0.95)
South Asia	227,459 (127,872 to 371,232)	43 (24.17 to 70.17)	366,728 (222,277 to 554,889)	36.43 (22.08 to 55.12)	61.23 (32.9 to 105.53)	−0.54 (−0.62 to −0.46)
Southeast Asia	126,605 (82,335 to 189,108)	53.51 (34.8 to 79.93)	209,051 (143,010 to 286,101)	56.38 (38.57 to 77.16)	65.12 (32.15 to 108.76)	0.41 (0.24 to 0.59)
Southern Latin America	17,249 (10,426 to 25,223)	70.43 (42.57 to 102.99)	14,078 (9,127 to 18,733)	40.59 (26.31 to 54)	−18.38 (−37.92 to 7.28)	−1.64 (−1.75 to −1.53)
Southern Sub-Saharan Africa	4,651 (3,188 to 6,501)	18.06 (12.38 to 25.24)	9,281 (6,197 to 13,341)	21.5 (14.35 to 30.9)	99.56 (57.09 to 147.87)	0.77 (0.37 to 1.16)
Tropical Latin America	76,302 (51,217 to 99,966)	97.18 (65.23 to 127.31)	76,946 (53,332 to 99,651)	64.21 (44.51 to 83.16)	0.84 (−9.26 to 12.18)	−1.71 (−1.9 to −1.52)
Western Europe	95,255 (68,105 to 120,029)	49.25 (35.21 to 62.06)	41,042 (28,689 to 52,433)	21.77 (15.22 to 27.81)	−56.91 (−60.61 to −53.03)	−2.79 (−2.99 to −2.6)
Western Sub-Saharan Africa	17,091 (7,085 to 42,688)	19.96 (8.28 to 49.87)	43,406 (20,011 to 103,753)	18.93 (8.73 to 45.25)	153.97 (90.49 to 233.59)	−0.02 (−0.19 to 0.15)

Geographically, high-income Asia Pacific, Western Europe, and Central Europe showed the largest reductions. High-income Asia Pacific deaths fell by 60.31% (95% UI −68.75 to −51.38) and DALYs by −58.19% (95% UI −66.74 to −49.12), with mortality and DALY EAPCs of −2.85% and −2.67% (Table 1 and Supplementary Table 1). Western Europe recorded similar trends, with deaths declining by 59.32% and DALYs by 56.91%, and EAPCs of −2.94% and −2.79%. Central Europe saw deaths decrease by 52.21% and DALYs by 49.89%, with mortality and DALY rates falling significantly (EAPC −2.09% and −1.89%) (Table 1 and Supplementary Table 1).

In contrast, regions such as Andean Latin America, the Caribbean, Oceania, and parts of Western Sub-Saharan Africa experienced large increases in both deaths and DALYs. For example, deaths rose by 169.73% in Oceania, 144.97% in the Andean Latin America, and 145.13% in Western Sub-Saharan Africa, accompanied by DALY increases of 173.17%, 148.71%, and 153.97%, respectively (Table 1 and Supplementary Table 1). Several of these regions also showed positive EAPCs in mortality or DALY rates, including Andean Latin America (mortality EAPC 1.35%), the Caribbean (mortality EAPC 0.93%), Oceania (DALY EAPC 0.75%), and Southern Sub-Saharan Africa (0.77%). Central Latin America and Southeast Asia reported increases in deaths and DALYs but relatively stable rates (Figures 2E, F). These patterns highlight persistent and widening disparities in SAH burden attributable to high SBP across global regions.

### National burden across 204 countries and territories

**FIGURE 3:**
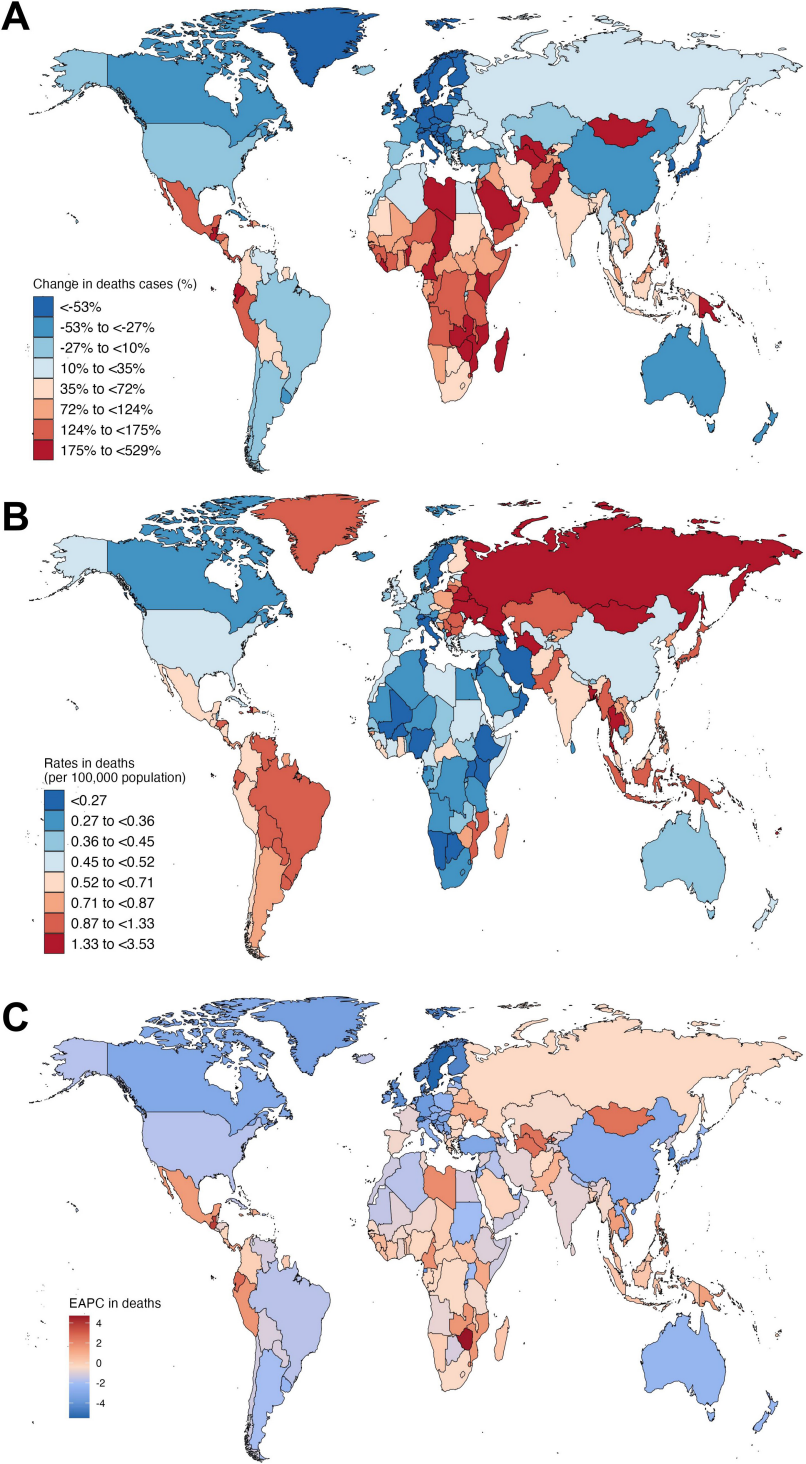
Global distribution of subarachnoid hemorrhage mortality attributable to high systolic blood pressure among young and middle-aged adults in 204 countries. **(A)** Change in the number of deaths from 1990 to 2021 by country. **(B)** Death rates (per 100,000 population) by country in 2021. **(C)** Estimated annual percentage change (EAPC) in death rates from 1990 to 2021 by country.

From 1990 to 2021, the mortality burden of SAH attributable to high SBP varied across 204 countries. The greatest increases in deaths were observed in Guatemala (529.40%, 95% UI 313.18 to 996.78), Uzbekistan (271.54%, 95% UI 161.55 to 446.59), and Ecuador (227.57%, 95% UI 100.11 to 549.26). In contrast, Slovenia (−74.44%, 95% UI −82.07 to −64.33), Sweden (−80.84%, 95% UI −86.23 to −74.02), and Croatia (−72.03%, 95% UI −79.35 to −61.82) reported the largest declines (Figure 3A and Supplementary Table 2). The highest mortality rates in 2021 were in Nauru (3.53 per 100,000, 95% UI 2.1 to 5.33), Haiti (2.62 per 100,000, 95% UI 1.15 to 4.39), and Vanuatu (2.56 per 100,000, 95% UI 1.35 to 4.42), while the lowest were in Jordan (0.06 per 100,000, 95% UI 0.04 to 0.1), Palestine (0.07 per 100,000, 95% UI 0.04 to 0.1), and Kuwait (0.08 per 100,000, 95% UI 0.05 to 0.01) (Figure 3B and Supplementary Table 2). Lesotho (EAPC 2.95%, 95% CI 2.46 to 3.45) and Guatemala (EAPC 3.76%, 95% CI 3.24 to 4.2) saw the steepest increases, while Sweden (−5.5%, 95% CI −5.75 to −5.25) and Singapore (−4.61%, 95% CI −4.98 to −4.22) achieved the largest reductions (Figure 3C and Supplementary Table 2).

From 1990 to 2021, SAH DALYs attributable to high SBP also showed large disparities. Guatemala (530.91%, 95% UI 321.09 to 993.11), Uzbekistan (267.52%, 95% UI 168.27 to 433.94), and Ecuador (223.60%, 95% UI 97.49 to 524.61) saw the largest increases, while Sweden (−76.58%, 95% UI −83.00 to −69.30), Estonia (−69.62%, 95% UI −77.14 to −59.91), and Croatia (−68.44%, 95% UI −76.32 to −57.76) reported the greatest declines (Supplementary Figure 1A and Supplementary Table 3). The highest DALY rates in 2021 were in Nauru (194.19 per 100,000, 95% UI 119.09 to 287.52), Vanuatu (142.39 per 100,000, 95% UI 80.55 to 238.57), and Haiti (134.64 per 100,000, 95% UI 60.6 to 223.66), while the lowest were in Jordan (6.33 per 100,000, 95% UI 3.86 to 9.37), Palestine (5.97 per 100,000, 95% UI 3.45 to 8.96), and Kuwait (8.2 per 100,000, 95% UI 5.19 to 11.79). Zimbabwe (EAPC 4.36%, 95% CI 3.53 to 5.19) and Guatemala (EAPC 3.84%, 95% CI 3.32 to 4.36) had the fastest increases, while Sweden (−4.98%, 95% CI −5.17 to −4.78) and Switzerland (−4.51%, 95% CI −4.79 to −4.23) saw the largest declines (Supplementary Figures 1B, C and Supplementary Table 3). These findings highlight growing inequalities in SAH mortality and DALY burden due to high SBP, with many low- and middle-income countries facing alarming increases, while high-income regions have made significant progress.

### Correlations between SAH burden and SDI

**FIGURE 4:**
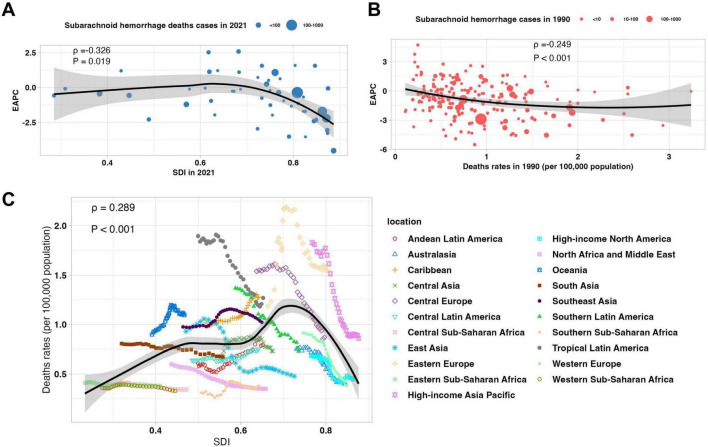
Correlations between subarachnoid hemorrhage mortality attributable to high systolic blood pressure, temporal trends, and socio-demographic development among young and middle-aged adults. **(A)** Association between the Socio-demographic Index (SDI) in 2021 and the estimated annual percentage change (EAPC) in death rates from 1990 to 2021. **(B)** Association between death rates in 1990 and EAPC in death rates from 1990 to 2021. **(C)** Relationship between SDI and death rates from 1990 to 2021 across global regions.

Globally, trends in SAH deaths attributable to high SBP varied across regions and SDI levels. As shown in Figure 4, there was a significant negative correlation between the EAPC of SAH death rates and SDI in 2021 (ρ = −0.326, *P* = 0.019), indicating that countries with higher SDI experienced more pronounced declines in mortality (Figure 4A). Similarly, a significant inverse correlation was observed between EAPC and the baseline SAH death rate in 1990 (ρ = −0.249, *P* < 0.001; Figure 4B), suggesting that countries with higher initial mortality achieved greater reductions over time. The relationship between SAH death rates and SDI followed a non-linear pattern: mortality rates increased with SDI up to approximately 0.65 and then declined as SDI rose further (ρ = 0.289, *P* < 0.001; Figure 4C), highlighting the disproportionate SAH mortality burden in middle-SDI countries, while many high-SDI regions have achieved substantial reductions. Temporal trends in SAH DALYs due to high SBP from 1990 to 2021 were also significantly associated with SDI levels and baseline burden. The EAPC in DALY rates was negatively correlated with SDI in 2021 (ρ = −0.343, *P* = 0.014), indicating that higher-SDI countries experienced greater reductions in DALYs (Supplementary Figure 2A). Similarly, a significant inverse association was observed between EAPC and DALY rates in 1990 (ρ = −0.274, *P* < 0.001; Supplementary Figure 2B), suggesting that regions with higher initial DALY burden achieved more pronounced declines. The relationship between SDI and DALY rates followed a similar non-linear pattern: DALY rates increased with SDI up to approximately 0.65, then declined as SDI continued to improve (ρ = 0.289, *P* < 0.001; Supplementary Figure 2C), emphasizing the disproportionate DALY burden in middle-SDI regions and the protective effect of higher SDI on SAH outcomes related to high SBP.

### Forecasted trends to 2050

**FIGURE 5:**
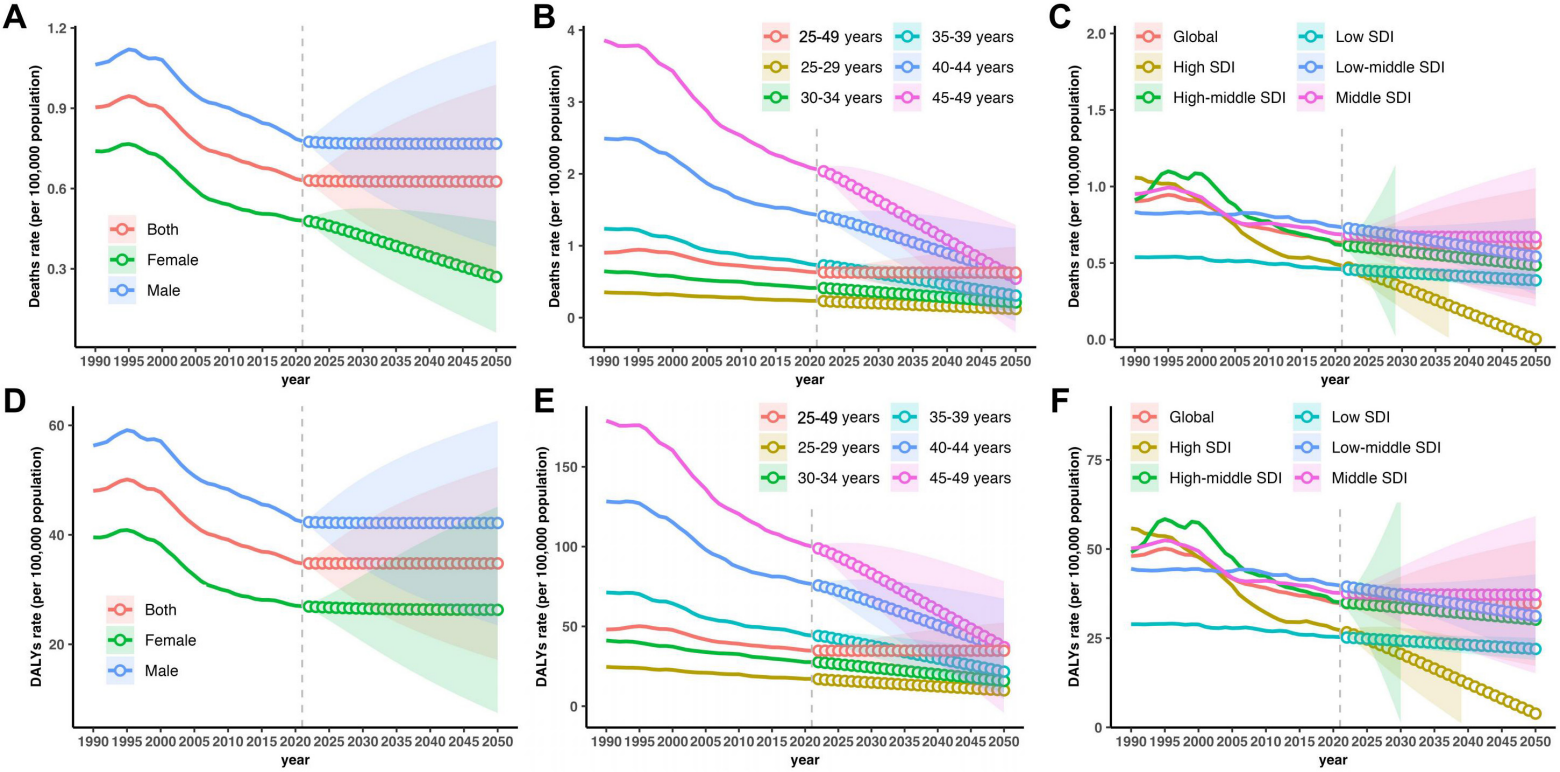
Projected subarachnoid hemorrhage deaths and disability-adjusted life years (DALYs) attributable to high systolic blood pressure among 25–49 years from 1990 to 2050, by sex, age and Socio-demographic Index (SDI) region. **(A–C)** Trends and projections in deaths rate by sex, age and SDI region. **(D–F)** Trends and projections in DALYs rate by sex, age and SDI region.

The forecasted trends in SAH deaths attributable to high SBP among individuals aged 25–49 years show continued global declines but substantial variation by sex, age, and region. By sex, SAH death rates in males are projected to plateau at approximately 0.77 (95% UI: 0.38–1.15) per 100,000 by 2050, while females are expected to achieve greater reductions from 0.46 (0.41–0.51) in 2025 to 0.27 (0.06–0.48) in 2050 (Figure 5A and Supplementary Table 4). Age-specific forecasts indicate more pronounced declines in older age groups: for example, deaths in the 45–49 age group are projected to fall from 1.90 (1.69–2.11) in 2025 to 0.54 (−0.21 to 1.29) by 2050. Globally, SAH death rates are expected to remain relatively stable at approximately 0.63 per 100,000 between 2025 and 2050, but with widening uncertainty intervals (2025: 0.56–0.69; 2050: 0.26–0.99) (Figure 5B and Supplementary Table 4). High-SDI regions are forecasted to achieve the greatest reductions, with death rates declining from 0.43 (0.36–0.50) to near-zero levels by 2050 (0.00, −0.30 to 0.31). Middle-SDI regions are projected to maintain rates around 0.67 (0.22–1.12), while low-SDI regions may experience a modest decline from 0.45 (0.43–0.47) to 0.39 (0.32–0.45) (Figure 5C and Supplementary Table 4). These projections highlight sustained progress in reducing SAH mortality linked to high SBP, particularly in high-SDI areas, though disparities across sexes, age groups, and regions are likely to persist.

The forecasted trends in SAH DALYs attributable to high SBP among individuals aged 25–49 years suggest a continued global decline, accompanied by persistent disparities by sex, age, and SDI level. By sex, male DALYs are projected to remain relatively stable at 42.16 (23.48–60.84) per 100,000 by 2050, whereas female DALYs may decline slightly from 26.72 (24.35–29.08) in 2025 to 26.30 (7.46–45.13) in 2050 (Figure 5D and Supplementary Table 4). Age-specific projections indicate sharper reductions among older subgroups, with DALYs in the 45–49 age group forecasted to drop from 93.67 (85.08–102.27) to 37.01 (−4.22 to 78.25). Globally, SAH DALY rates are expected to stabilize at around 34.78 per 100,000 between 2025 and 2050, with progressively wider uncertainty intervals (2025: 31.53–38.03; 2050: 17.17–52.38) (Figure 5E and Supplementary Table 4). High-SDI regions are forecasted to achieve the most substantial reductions, from 24.61 (21.09–28.14) to 3.86 (−12.19 to 19.91), while high-middle SDI regions may experience less consistent declines, with DALYs projected at 30.18 (−146.58 to 206.94) by 2050. Middle-SDI and low-middle SDI regions are expected to have modest reductions or stable rates, at 37.19 (15.18–59.19) and 31.20 (19.49–42.90), respectively. Low-SDI regions are projected to see a gradual decline to 21.97 (18.87–25.07) (Figure 5F and Supplementary Table 4). These forecasts underscore global progress in reducing SAH DALYs related to high SBP, particularly in high-SDI settings, while emphasizing the need for targeted strategies to address ongoing inequities.

## Discussion

This study provides a comprehensive assessment of global and regional trends in SAH mortality and DALYs attributable to high SBP among adults aged 25–49 years, along with projections to 2050. The findings highlight substantial progress in reducing the burden of SAH associated with elevated SBP, particularly in high-SDI regions, while underscoring persistent disparities across socio-demographic, sex, and age groups.

Moreover, clear sex-specific disparities were observed. Males consistently exhibited higher mortality and DALYs attributable to high SBP-related SAH than females, which may be associated with poorer hypertension control, higher prevalence of smoking and alcohol consumption, and lower healthcare utilization in men. In contrast, the relative decline in female burden may relate to improved awareness and management of blood pressure, as well as protective hormonal factors before menopause. These findings align with previous epidemiological studies reporting sex differences in stroke and SAH outcomes (Franssen et al., 2025; Zhang C. et al., 2025; Zhang J. et al., 2025).

The significant decline in SAH mortality and DALYs in high-SDI regions aligns with decades of investment in hypertension screening, treatment, and stroke care. Prior studies have shown that improved hypertension control, widespread availability of antihypertensive medications, and the establishment of organized stroke systems of care, including aneurysm screening and early surgical or endovascular intervention, have contributed to substantial reductions in SAH mortality in these settings (Boumiza et al., 2021; Nave et al., 2014; Ota and Matsubara, 2021). Moreover, the decline in case fatality rates may reflect advances in neurocritical care and rehabilitation services. Mechanistically, these trends may relate to better control of chronic vascular damage induced by sustained high SBP, including reduced progression of cerebral aneurysms and arterial dissections, both key pathological substrates of SAH (Cayron et al., 2025; Pinsky et al., 2024; Schmidt et al., 2021). Experimental and clinical studies have also suggested that effective SBP management can modulate vessel wall remodeling and decrease the risk of aneurysm rupture by limiting matrix degradation, smooth muscle cell apoptosis, and inflammatory infiltration in cerebral arteries (Chi et al., 2025; Jeong et al., 2025; Lussier et al., 2024).

By contrast, many middle- and low-SDI regions have seen slower progress or even increases in SAH burden. These trends reflect ongoing gaps in hypertension detection, access to affordable care, and population-level prevention strategies. In several of these regions, structural barriers such as weak primary care systems, inadequate health financing, and limited availability of essential medications hinder effective blood pressure management (Papatsoris et al., 2025). Additionally, population transitions toward urbanization, dietary shifts toward high sodium and low potassium intake, and rising prevalence of obesity have further exacerbated hypertension-related risks (Jozwiak et al., 2025; Knowles and Raitt, 2025). Notably, the widening uncertainty intervals in our projections, particularly in middle-SDI settings, highlight the critical importance of strengthening surveillance data quality to better guide policy responses.

Age-specific patterns reveal that SAH burden increases progressively across the 25–49 age range, peaking in the 45–49 group, consistent with the cumulative vascular injury associated with prolonged exposure to elevated SBP. The steeper declines in burden within this age band likely reflect targeted hypertension interventions among older working-age adults, as well as secondary prevention efforts in individuals identified as high risk (Ofori et al., 2024). Looking ahead, the projected continued decline in SAH burden globally—especially in high-SDI regions—is encouraging, yet the persistence of stark disparities underscores the need for intensified efforts in low- and middle-SDI settings. Multifaceted strategies are essential, combining population-level measures (such as salt reduction, promotion of healthy diets, tobacco and alcohol control) with expanded access to hypertension screening, diagnosis, and treatment (Gu et al., 2025). Integration of cardiovascular risk management into universal health coverage and primary care systems represents a key opportunity to address these challenges. Furthermore, emerging evidence on the molecular mechanisms linking SBP to aneurysm pathogenesis—such as the roles of transforming growth factor-β signaling, matrix metalloproteinases, and inflammatory cytokines—highlights the potential for future pharmacological interventions beyond blood pressure control alone (Glass et al., 2014).

Several limitations should be noted. The accuracy of our estimates depends on the quality and availability of primary data, which remain limited in some low-SDI regions. In addition, projections are based on current trends and assume no major disruptions, but future policy shifts, technological innovations, or unforeseen events (e.g., pandemics) could alter trajectories (GBD 2021 Causes of Death Collaborators, 2024; Murray and GBD 2021 Collaborators, 2024). Despite these uncertainties, the study offers valuable insights to inform global and regional strategies aimed at reducing the preventable burden of SAH attributable to high SBP.

## Conclusion

In conclusion, despite global declines in SAH mortality and DALYs attributable to high SBP, large disparities remain across sex, age, and SDI levels. These findings highlight the urgent need for equitable hypertension control and strengthened health systems, particularly in low- and middle-SDI regions. Advances in understanding the vascular effects of high SBP and aneurysm pathophysiology may offer new prevention strategies. Focused, data-driven interventions are essential to further reduce premature SAH deaths and disability worldwide.

## Data Availability

The original contributions presented in this study are included in this article/Supplementary material, further inquiries can be directed to the corresponding author.

## Footnotes

1
https://ghdx.healthdata.org/gbd-2021
