# Supplementary material for: Rising burden of subarachnoid hemorrhage linked to high systolic blood pressure among young and middle-aged populations: temporal trends and global implication
Source: Front Hum Neurosci. 2025 Oct 30;19:1700918. doi: 10.3389/fnhum.2025.1700918 (PMC12611887; doi:10.3389/fnhum.2025.1700918)
Supplement: Supplementary file 1 [file Data_Sheet_1.pdf]

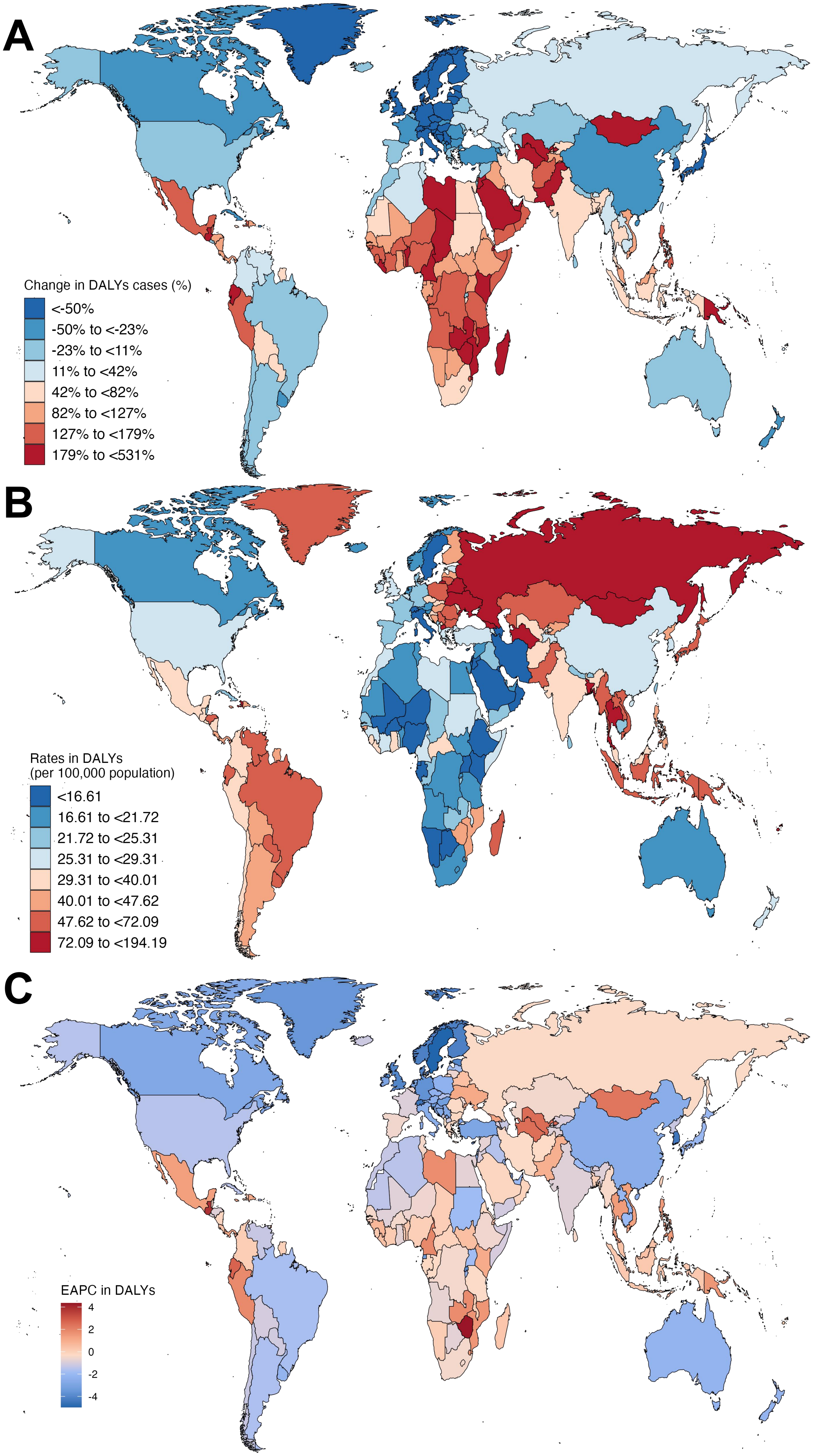

Supplementary Figure S1. Global distribution of subarachnoid hemorrhage mortality attributable to high systolic blood pressure among young and middle-aged adults in 204 countries.

(A) Change in the number of deaths from 1990 to 2021 by country.

(B) Death rates (per 100,000 population) by country in 2021.

(C) EAPC in death rates from 1990 to 2021 by country.

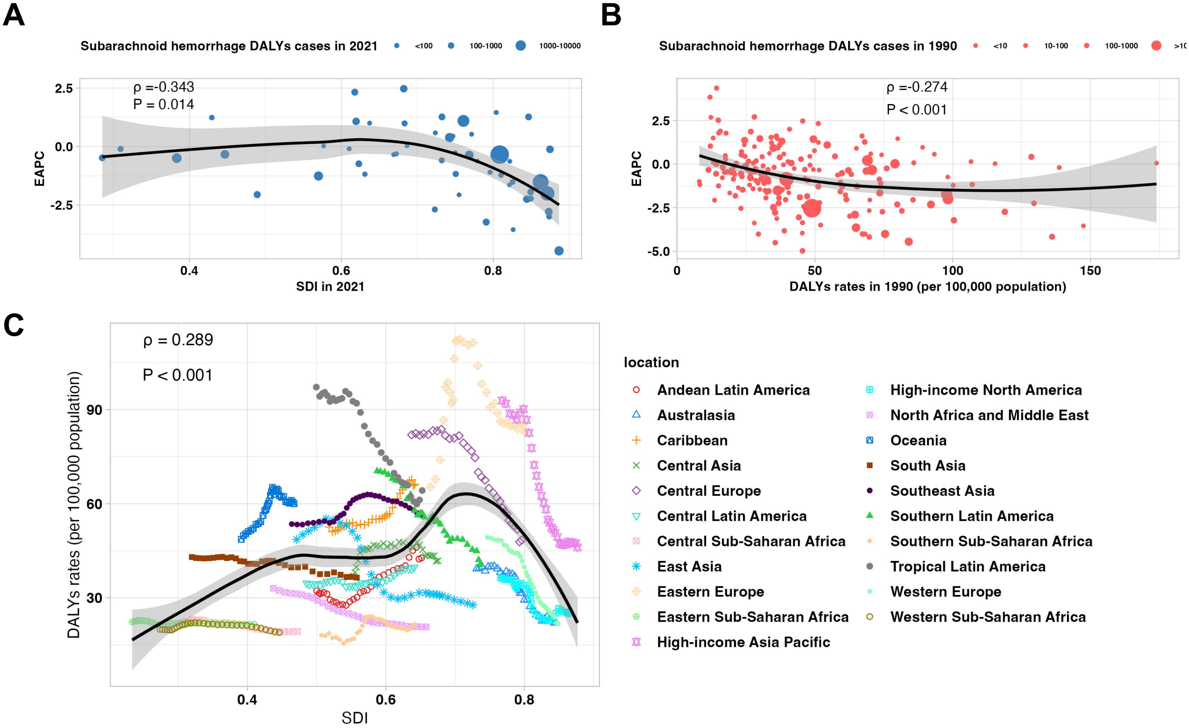

Supplementary Figure S2. Correlations between subarachnoid hemorrhage DALYs attributable to high systolic blood pressure, temporal trends, and socio-demographic development among young and middle-aged adults.
(A) Association between the SDI in 2021 and the EAPC in DALYs rate from 1990 to 2021.
(B) Association between DALYs rate in 1990 and EAPC in DALYs rate from 1990 to 2021.
(C) Relationship between SDI and DALYs rate from 1990 to 2021 across global regions.

Supplementary Table S1. Global and regional DALYs and trends of subarachnoid hemorrhage attributable to high systolic blood pressure among 25-49 aged from 1990 to 2021.

Feature	Cases_1990	Rates_1990	Cases_2021	Rates_2021	Cases_change	EAPC_CI
**Global**	1301770 (844050 to 1811022)	48.03 (31.14 to 66.82)	1373366 (910290 to 1871996)	34.78 (23.05 to 47.41)	5.5 (-9.75 to 22.3)	-1.31 (-1.42 to -1.19)
**Sex group**						
Female	528734 (329828 to 738033)	39.54 (24.66 to 55.19)	525577 (353937 to 716231)	26.97 (18.16 to 36.75)	-0.6 (-17.94 to 27.97)	-1.59 (-1.73 to -1.45)
Male	773036 (470516 to 1150603)	56.3 (34.27 to 83.8)	847789 (567158 to 1224213)	42.4 (28.36 to 61.22)	9.67 (-7.94 to 35.54)	-1.12 (-1.23 to -1.02)
**Age group**						
25-29 years	109155 (62420 to 174314)	24.66 (14.1 to 39.38)	100874 (60450 to 157823)	17.15 (10.27 to 26.82)	-7.59 (-27.08 to 18.43)	-1.27 (-1.32 to -1.23)
30-34 years	158747 (94880 to 234989)	41.19 (24.62 to 60.97)	167210 (103985 to 245806)	27.66 (17.2 to 40.66)	5.33 (-14.82 to 31.54)	-1.35 (-1.39 to -1.32)
35-39 years	250989 (152784 to 367996)	71.25 (43.37 to 104.47)	248286 (160751 to 346138)	44.27 (28.66 to 61.71)	-1.08 (-21.18 to 25.56)	-1.69 (-1.77 to -1.6)
40-44 years	367606 (239871 to 524965)	128.32 (83.73 to 183.25)	382904 (250805 to 529126)	76.54 (50.14 to 105.77)	4.16 (-15.03 to 27.66)	-1.97 (-2.09 to -1.85)
45-49 years	415274 (269892 to 574802)	178.85 (116.23 to 247.55)	474092 (327657 to 634374)	100.12 (69.2 to 133.97)	14.16 (-5.68 to 40.14)	-2.15 (-2.26 to -2.04)
**SDI regions**						
High SDI	257149 (179546 to 332304)	55.8 (38.96 to 72.11)	136726 (90386 to 179979)	27.22 (18 to 35.84)	-46.83 (-51.74 to -42.42)	-2.69 (-2.87 to -2.51)
High-middle SDI	277247 (177049 to 373706)	49.12 (31.37 to 66.21)	220909 (153390 to 282799)	35.09 (24.36 to 44.92)	-20.32 (-35.04 to -1.13)	-1.67 (-1.93 to -1.4)
Middle SDI	456959 (268870 to 667106)	50.18 (29.53 to 73.26)	472948 (320255 to 623500)	37.68 (25.52 to 49.68)	3.5 (-19.32 to 36.39)	-1.18 (-1.32 to -1.05)
Low-middle	244975	44.45	404020	39.76	64.92 (40.24	-0.31 (-

Feature	Cases_1990	Rates_1990	Cases_2021	Rates_2021	Cases_change	EAPC_CI
SDI	(147577 to 375134)	(26.78 to 68.07)	(256757 to 602961)	(25.27 to 59.33)	to 97.89)	0.38 to -0.24)
Low SDI	63896 (31487 to 122936)	28.91 (14.24 to 55.62)	137230 (66770 to 288291)	25.3 (12.31 to 53.15)	114.77 (73.94 to 163.35)	-0.48 (-0.53 to -0.43)
**Geographical regions**						
Andean Latin America	6012 (2832 to 9694)	32.26 (15.2 to 52.02)	14952 (9084 to 21790)	42.75 (25.97 to 62.3)	148.71 (67.8 to 295.25)	1.43 (1.11 to 1.75)
Australasia	4239 (2860 to 5613)	39.28 (26.5 to 52.01)	3179 (1944 to 4386)	22.02 (13.46 to 30.37)	-25 (-38.98 to -11.23)	-2.24 (-2.47 to -2.02)
Caribbean	9603 (5849 to 13783)	52.57 (32.02 to 75.45)	15854 (9478 to 23271)	66.21 (39.58 to 97.19)	65.09 (22.15 to 132.11)	0.94 (0.81 to 1.06)
Central Asia	12321 (8446 to 15652)	36.95 (25.33 to 46.94)	20376 (14634 to 26339)	41.79 (30.01 to 54.02)	65.37 (36.17 to 100.75)	0.16 (-0.08 to 0.4)
Central Europe	50907 (36665 to 64017)	81.98 (59.05 to 103.09)	25510 (18482 to 31664)	48.42 (35.08 to 60.09)	-49.89 (-55.2 to -43.83)	-1.89 (-2.09 to -1.69)
Central Latin America	28252 (18262 to 38165)	34.61 (22.37 to 46.76)	52879 (34787 to 71251)	39.72 (26.13 to 53.52)	87.17 (54.83 to 132.43)	0.42 (0.29 to 0.54)
Central Sub-Saharan Africa	5310 (2380 to 11203)	21.75 (9.75 to 45.88)	12582 (4945 to 34615)	19.3 (7.58 to 53.09)	136.96 (53.42 to 284.14)	-0.54 (-0.68 to -0.39)
East Asia	334938 (157284 to 539980)	48.62 (22.83 to 78.39)	190938 (112361 to 276511)	27.73 (16.32 to 40.16)	-42.99 (-64.43 to -3.83)	-2.47 (-2.87 to -2.06)
Eastern Europe	72060 (52546 to 88658)	65.34 (47.64 to 80.39)	79953 (56724 to 98777)	83.09 (58.95 to 102.65)	10.95 (-0.21 to 22.48)	-0.08 (-0.61 to 0.46)
Eastern Sub-Saharan Africa	18610 (6439 to 47708)	22.31 (7.72 to 57.19)	45075 (16155 to 125154)	21.53 (7.71 to 59.77)	142.21 (94.43 to 202.85)	-0.15 (-0.21 to -0.1)
High-income Asia Pacific	86230 (60504 to 110274)	92.9 (65.18 to 118.8)	36052 (23784 to 47888)	46.09 (30.41 to 61.22)	-58.19 (-66.74 to -49.12)	-2.67 (-2.95 to -2.39)
High-income	54214	36.38	42004	24.9 (15.3	-22.52 (-35.45	-1.62 (-

Feature	Cases_1990	Rates_1990	Cases_2021	Rates_2021	Cases_change	EAPC_CI
North America	(35052 to 73781)	(23.52 to 49.51)	(25812 to 57826)	to 34.28)	to -7)	1.93 to -1.3)
North Africa and Middle East	52908 (32295 to 77935)	33.01 (20.15 to 48.63)	69232 (44110 to 97699)	20.71 (13.19 to 29.22)	30.86 (-9.19 to 68.43)	-1.67 (-1.79 to -1.55)
Oceania	1554 (825 to 2629)	48.65 (25.81 to 82.29)	4246 (2268 to 7129)	60.02 (32.05 to 100.77)	173.17 (91.3 to 293.52)	0.75 (0.54 to 0.95)
South Asia	227459 (127872 to 371232)	43 (24.17 to 70.17)	366728 (222277 to 554889)	36.43 (22.08 to 55.12)	61.23 (32.9 to 105.53)	-0.54 (-0.62 to -0.46)
Southeast Asia	126605 (82335 to 189108)	53.51 (34.8 to 79.93)	209051 (143010 to 286101)	56.38 (38.57 to 77.16)	65.12 (32.15 to 108.76)	0.41 (0.24 to 0.59)
Southern Latin America	17249 (10426 to 25223)	70.43 (42.57 to 102.99)	14078 (9127 to 18733)	40.59 (26.31 to 54)	-18.38 (-37.92 to 7.28)	-1.64 (-1.75 to -1.53)
Southern Sub-Saharan Africa	4651 (3188 to 6501)	18.06 (12.38 to 25.24)	9281 (6197 to 13341)	21.5 (14.35 to 30.9)	99.56 (57.09 to 147.87)	0.77 (0.37 to 1.16)
Tropical Latin America	76302 (51217 to 99966)	97.18 (65.23 to 127.31)	76946 (53332 to 99651)	64.21 (44.51 to 83.16)	0.84 (-9.26 to 12.18)	-1.71 (-1.9 to -1.52)
Western Europe	95255 (68105 to 120029)	49.25 (35.21 to 62.06)	41042 (28689 to 52433)	21.77 (15.22 to 27.81)	-56.91 (-60.61 to -53.03)	-2.79 (-2.99 to -2.6)
Western Sub-Saharan Africa	17091 (7085 to 42688)	19.96 (8.28 to 49.87)	43406 (20011 to 103753)	18.93 (8.73 to 45.25)	153.97 (90.49 to 233.59)	-0.02 (-0.19 to 0.15)

Supplementary Table S2. Trends in subarachnoid hemorrhage deaths attributable to high systolic blood pressure in 204 countries from 1990 to 2021.

Country	Cases_1990	Rates_1990	Cases_2021	Rates_2021	Cases_change	EAPC_CI
American Samoa	0 (0 to 0)	1.22 (0.72 to 1.76)	0 (0 to 1)	1.59 (0.94 to 2.38)	28.74 (-22.36 to 104.92)	0.73 (0.56 to 0.9)
Antigua and Barbuda	0 (0 to 0)	0.79 (0.44 to 1.16)	0 (0 to 0)	0.57 (0.34 to 0.81)	8.99 (-30.08 to 67.57)	-0.21 (-0.5 to 0.09)
Arab Republic of Egypt	129 (55 to 232)	0.48 (0.2 to 0.87)	166 (92 to 283)	0.31 (0.17 to 0.53)	28.67 (-27.69 to 158.8)	-1.18 (-1.31 to -1.05)
Argentine Republic	230 (128 to 354)	1.45 (0.81 to 2.23)	172 (109 to 241)	0.73 (0.46 to 1.02)	-25 (-48.93 to 13.49)	-1.97 (-2.17 to -1.78)
Australia	63 (42 to 84)	0.7 (0.47 to 0.94)	46 (28 to 65)	0.38 (0.23 to 0.54)	-26.86 (-43.58 to -7.4)	-2.41 (-2.69 to -2.12)
Barbados	1 (1 to 2)	0.92 (0.57 to 1.28)	1 (1 to 2)	1.03 (0.62 to 1.48)	15.9 (-20.59 to 68.2)	0.52 (0.33 to 0.71)
Belize	0 (0 to 1)	0.44 (0.24 to 0.68)	1 (1 to 1)	0.44 (0.25 to 0.63)	177.38 (79.68 to 347.76)	-0.04 (-0.27 to 0.19)
Bermuda	0 (0 to 0)	0.62 (0.34 to 0.94)	0 (0 to 0)	0.38 (0.21 to 0.55)	-50.88 (-69.63 to -20.42)	-1.7 (-1.95 to -1.46)
Bolivarian Republic of Venezuela	116 (80 to 150)	1.21 (0.84 to 1.57)	149 (82 to 230)	1.13 (0.62 to 1.75)	28.5 (-17.15 to 82.27)	-1.22 (-1.75 to -0.7)
Bosnia and Herzegovina	37 (23 to 52)	1.53 (0.97 to 2.18)	11 (6 to 16)	0.74 (0.43 to 1.1)	-69.98 (-82.3 to -52.19)	-2.96 (-3.31 to -2.6)
Brunei Darussalam	3 (2 to 4)	1.91 (1.1 to 2.86)	4 (2 to 6)	1.46 (0.91 to 2.07)	41.22 (-7.75 to 122.26)	-1.13 (-1.53 to -0.73)
Burkina Faso	12 (3 to 33)	0.31 (0.09 to 0.87)	27 (7 to 71)	0.26 (0.07 to 0.69)	123.57 (38.89 to 291.98)	-0.57 (-0.74 to -0.41)
Canada	95 (60 to 131)	0.65 (0.41 to 0.89)	50 (28 to 77)	0.3 (0.17 to 0.46)	-47.04 (-62.08 to -28.03)	-3.05 (-3.37 to -2.74)
Central	7 (3 to 17)	0.59 (0.22	16 (4 to 40)	0.59 (0.16	112.33 (14.36	-0.08 (-

Country	Cases_1990	Rates_1990	Cases_2021	Rates_2021	Cases_change	EAPC_CI
African Republic		to 1.41)		to 1.49)	to 273.69)	0.16 to -0.01)
Commonwealth of Dominica	0 (0 to 0)	0.64 (0.36 to 0.99)	0 (0 to 0)	0.75 (0.45 to 1.15)	11.79 (-30.22 to 75.05)	0.68 (0.53 to 0.84)
Commonwealth of the Bahamas	1 (1 to 1)	0.72 (0.42 to 1.02)	2 (1 to 3)	0.87 (0.53 to 1.28)	76.37 (9.58 to 196.02)	0.74 (0.53 to 0.95)
Cook Islands	0 (0 to 0)	1.04 (0.49 to 1.86)	0 (0 to 0)	0.7 (0.4 to 1.15)	-42.83 (-71.48 to 25.07)	-0.95 (-1.14 to -0.75)
Czech Republic	78 (58 to 99)	1.5 (1.11 to 1.9)	25 (16 to 34)	0.53 (0.35 to 0.72)	-68.03 (-75.81 to -59.07)	-2.77 (-3.05 to -2.49)
Democratic People's Republic of Korea	120 (62 to 208)	1.12 (0.58 to 1.94)	119 (52 to 247)	0.86 (0.38 to 1.79)	-1.24 (-50.86 to 103.27)	-1.21 (-1.38 to -1.04)
Democratic Republic of Sao Tome and Principe	0 (0 to 1)	0.42 (0.15 to 1.13)	1 (0 to 2)	0.6 (0.19 to 1.74)	221.11 (82.09 to 457.79)	1.14 (0.75 to 1.54)
Democratic Republic of the Congo	60 (21 to 145)	0.36 (0.13 to 0.86)	142 (36 to 512)	0.33 (0.08 to 1.18)	135.63 (11.19 to 347.97)	-0.43 (-0.63 to -0.23)
Democratic Republic of Timor-Leste	3 (2 to 6)	0.87 (0.44 to 1.59)	6 (3 to 11)	0.84 (0.44 to 1.63)	72.1 (-1.23 to 208.5)	-0.06 (-0.43 to 0.31)
Democratic Socialist Republic of Sri Lanka	38 (21 to 61)	0.41 (0.23 to 0.66)	33 (18 to 51)	0.3 (0.16 to 0.47)	-13.44 (-51.6 to 47.26)	-0.83 (-1.06 to -0.59)
Dominican Republic	25 (13 to 41)	0.7 (0.36 to 1.13)	48 (26 to 75)	0.83 (0.44 to 1.28)	90.76 (9.7 to 236.96)	1.14 (0.92 to 1.36)
Eastern Republic of Uruguay	37 (23 to 53)	2.5 (1.52 to 3.57)	22 (14 to 29)	1.32 (0.84 to 1.79)	-41.7 (-58.97 to -15.24)	-2.33 (-2.56 to -2.1)
Federal Democratic Republic of Ethiopia	58 (12 to 173)	0.27 (0.05 to 0.8)	109 (30 to 339)	0.2 (0.05 to 0.62)	87.82 (18.15 to 215.51)	-1.06 (-1.4 to -0.72)
Federal Democratic	74 (35 to	0.84 (0.4 to	77 (33 to	0.46 (0.2 to	3.07 (-43.56	-1.9 (-2.02 to -

Country	Cases_1990	Rates_1990	Cases_2021	Rates_2021	Cases_change	EAPC_CI
Republic of Nepal	138)	1.55)	143)	0.86)	to 77.35)	1.77)
Federal Republic of Germany	479 (336 to 609)	1.2 (0.84 to 1.53)	138 (88 to 184)	0.39 (0.25 to 0.52)	-71.19 (-77.88 to -63.23)	-3.77 (-4.01 to -3.52)
Federal Republic of Nigeria	103 (33 to 298)	0.25 (0.08 to 0.73)	231 (86 to 662)	0.21 (0.08 to 0.61)	123.14 (34.96 to 270.1)	-0.32 (-0.54 to -0.1)
Federal Republic of Somalia	22 (4 to 73)	0.63 (0.11 to 2.09)	49 (7 to 191)	0.5 (0.07 to 1.92)	126.27 (0.88 to 358.51)	-1.28 (-1.59 to -0.98)
Federated States of Micronesia	1 (0 to 2)	2 (0.94 to 3.52)	1 (1 to 2)	2.1 (1.04 to 3.59)	19.32 (-30.66 to 118.04)	0.09 (0.03 to 0.15)
Federative Republic of Brazil	1462 (982 to 1909)	1.91 (1.28 to 2.49)	1482 (1034 to 1922)	1.28 (0.89 to 1.66)	1.36 (-8.89 to 13.52)	-1.69 (-1.91 to -1.47)
French Republic	170 (115 to 220)	0.58 (0.39 to 0.75)	121 (79 to 165)	0.43 (0.28 to 0.58)	-28.63 (-46.96 to -10.18)	-0.87 (-1.3 to -0.44)
Gabonese Republic	1 (1 to 3)	0.29 (0.13 to 0.57)	3 (1 to 5)	0.28 (0.12 to 0.54)	102.51 (14.72 to 291.37)	-0.29 (-0.57 to -0.02)
Georgia	32 (21 to 43)	1.18 (0.77 to 1.59)	28 (18 to 38)	1.74 (1.15 to 2.39)	-11.85 (-41.43 to 30.88)	1.57 (0.98 to 2.15)
Grand Duchy of Luxembourg	2 (1 to 2)	0.84 (0.56 to 1.12)	1 (0 to 1)	0.23 (0.15 to 0.31)	-56.63 (-68.65 to -39.1)	-4.33 (-4.7 to -3.95)
Greenland	1 (1 to 2)	2.93 (1.59 to 4.68)	0 (0 to 0)	1.05 (0.48 to 1.7)	-71.77 (-84.53 to -51.32)	-3.53 (-3.91 to -3.15)
Grenada	0 (0 to 1)	0.97 (0.54 to 1.43)	0 (0 to 1)	0.84 (0.51 to 1.2)	18.2 (-23.68 to 92.76)	-0.25 (-0.51 to 0)
Guam	0 (0 to 1)	0.6 (0.31 to 0.96)	1 (0 to 1)	1.05 (0.66 to 1.49)	69.92 (10.27 to 214.05)	1.59 (1.31 to 1.87)
Guyana	4 (2 to 6)	0.96 (0.55 to 1.44)	5 (3 to 7)	1.19 (0.69 to 1.79)	22.8 (-19.98 to 95.22)	0.87 (0.52 to 1.23)
Hashemite Kingdom of	2 (1 to 3)	0.11 (0.06 to 0.18)	4 (2 to 7)	0.06 (0.04 to 0.1)	123.77 (19.28 to 308.19)	-2.11 (-2.29 to -

Country	Cases_1990	Rates_1990	Cases_2021	Rates_2021	Cases_change	EAPC_CI
Jordan						1.94)
Hellenic Republic	25 (16 to 35)	0.5 (0.32 to 0.69)	21 (12 to 30)	0.48 (0.27 to 0.68)	-18.45 (-39.97 to 10.56)	-0.23 (-0.43 to -0.03)
Hungary	97 (70 to 120)	1.91 (1.38 to 2.36)	32 (21 to 42)	0.73 (0.48 to 0.97)	-67.52 (-75.43 to -58.6)	-3.52 (-3.74 to -3.29)
Independent State of Papua New Guinea	13 (5 to 26)	0.66 (0.26 to 1.33)	51 (23 to 96)	0.96 (0.42 to 1.79)	293.5 (115.44 to 718.3)	1.41 (1.07 to 1.75)
Independent State of Samoa	1 (0 to 2)	1.17 (0.56 to 2.05)	2 (1 to 3)	1.56 (0.81 to 2.68)	70.41 (-1.93 to 188.88)	0.86 (0.67 to 1.05)
Ireland	21 (14 to 26)	1.16 (0.79 to 1.48)	10 (7 to 13)	0.44 (0.29 to 0.58)	-51.25 (-64 to -36.5)	-3.27 (-3.61 to -2.93)
Islamic Republic of Afghanistan	37 (13 to 72)	0.91 (0.32 to 1.79)	93 (35 to 191)	0.63 (0.24 to 1.3)	154.61 (32.61 to 388.47)	-0.4 (-0.83 to 0.04)
Islamic Republic of Iran	49 (29 to 76)	0.19 (0.11 to 0.3)	67 (41 to 107)	0.14 (0.09 to 0.23)	37.39 (-18.96 to 89.55)	-0.9 (-1.19 to -0.6)
Islamic Republic of Mauritania	5 (2 to 12)	0.49 (0.18 to 1.29)	6 (2 to 20)	0.31 (0.11 to 0.95)	43.17 (-21.6 to 144.31)	-1.55 (-1.66 to -1.45)
Islamic Republic of Pakistan	317 (174 to 533)	0.64 (0.35 to 1.08)	1073 (598 to 1754)	0.88 (0.49 to 1.44)	238.39 (126.98 to 394.75)	0.83 (0.7 to 0.96)
Jamaica	8 (4 to 12)	0.67 (0.36 to 1.01)	13 (7 to 19)	0.82 (0.46 to 1.21)	60.93 (7.35 to 159.44)	1.11 (0.74 to 1.48)
Japan	1257 (906 to 1541)	1.94 (1.4 to 2.37)	540 (353 to 718)	1.07 (0.7 to 1.42)	-57.01 (-65.84 to -48.03)	-2.2 (-2.51 to -1.89)
Kingdom of Bahrain	1 (1 to 1)	0.3 (0.18 to 0.48)	2 (1 to 3)	0.21 (0.13 to 0.33)	124.69 (28.54 to 259.46)	-2 (-2.37 to -1.64)
Kingdom of Belgium	29 (18 to 39)	0.58 (0.37 to 0.78)	24 (16 to 31)	0.48 (0.33 to 0.63)	-16.48 (-33.34 to 6.64)	-0.76 (-1.3 to -0.22)
Kingdom of Bhutan	2 (1 to 3)	0.53 (0.23 to 1.05)	2 (1 to 3)	0.45 (0.22 to 0.79)	18.83 (-35.06 to 116.15)	-1.09 (-1.26 to -0.92)

Country	Cases_1990	Rates_1990	Cases_2021	Rates_2021	Cases_change	EAPC_CI
Kingdom of Cambodia	32 (14 to 57)	0.69 (0.32 to 1.25)	37 (17 to 69)	0.41 (0.19 to 0.76)	16.14 (-39.49 to 127.52)	-2.12 (-2.33 to -1.91)
Kingdom of Denmark	34 (24 to 44)	1.29 (0.9 to 1.65)	9 (6 to 13)	0.36 (0.23 to 0.49)	-72.75 (-79.86 to -64.73)	-4.37 (-4.91 to -3.84)
Kingdom of Eswatini	1 (0 to 2)	0.23 (0.11 to 0.47)	2 (1 to 4)	0.34 (0.16 to 0.66)	153.49 (31.24 to 392.22)	1.62 (1.01 to 2.22)
Kingdom of Lesotho	1 (0 to 3)	0.21 (0.07 to 0.5)	4 (2 to 9)	0.44 (0.2 to 0.86)	213.94 (56.14 to 572.13)	2.95 (2.46 to 3.45)
Kingdom of Morocco	89 (36 to 159)	0.71 (0.29 to 1.28)	97 (44 to 184)	0.5 (0.23 to 0.95)	10 (-34.14 to 102.08)	-1.31 (-1.47 to -1.16)
Kingdom of Norway	29 (20 to 35)	1.32 (0.94 to 1.64)	8 (6 to 11)	0.33 (0.23 to 0.42)	-71.37 (-76.97 to -66.03)	-4.56 (-4.85 to -4.27)
Kingdom of Saudi Arabia	23 (13 to 37)	0.28 (0.16 to 0.46)	70 (33 to 120)	0.28 (0.13 to 0.47)	208.66 (34.63 to 525.56)	-0.15 (-0.32 to 0.02)
Kingdom of Spain	81 (51 to 110)	0.42 (0.26 to 0.57)	76 (50 to 104)	0.38 (0.25 to 0.52)	-5.63 (-29.29 to 27.55)	-0.6 (-0.99 to -0.2)
Kingdom of Sweden	36 (24 to 47)	0.87 (0.58 to 1.13)	7 (4 to 11)	0.15 (0.09 to 0.24)	-80.84 (-86.22 to -74.02)	-5.5 (-5.75 to -5.25)
Kingdom of Thailand	297 (158 to 499)	0.94 (0.5 to 1.58)	488 (277 to 792)	1.53 (0.87 to 2.48)	64.44 (-8.71 to 176.41)	1.48 (1.07 to 1.89)
Kingdom of the Netherlands	66 (42 to 90)	0.81 (0.52 to 1.11)	23 (15 to 31)	0.31 (0.2 to 0.42)	-65.77 (-74.47 to -52.34)	-3.34 (-3.87 to -2.81)
Kingdom of Tonga	0 (0 to 0)	0.61 (0.32 to 1.1)	0 (0 to 1)	0.73 (0.42 to 1.29)	31.92 (-18.42 to 115.66)	0.85 (0.71 to 0.99)
Kyrgyz Republic	18 (10 to 26)	0.86 (0.48 to 1.26)	30 (17 to 45)	0.87 (0.5 to 1.3)	64.29 (1.15 to 168.4)	-0.89 (-1.5 to -0.27)
Lao People's Democratic Republic	31 (15 to 54)	1.65 (0.8 to 2.89)	35 (20 to 56)	0.87 (0.49 to 1.41)	13.56 (-35.31 to 117.69)	-2.29 (-2.38 to -2.19)
Lebanon	14 (7 to 23)	0.97 (0.52 to 1.6)	10 (7 to 15)	0.34 (0.22 to 0.49)	-26.7 (-58.16 to 28.31)	-3.56 (-4.2 to -

Country	Cases_1990	Rates_1990	Cases_2021	Rates_2021	Cases_change	EAPC_CI
						2.93)
Malaysia	56 (34 to 83)	0.62 (0.38 to 0.93)	99 (66 to 139)	0.56 (0.37 to 0.78)	78.57 (13.71 to 179.87)	0.24 (-0.14 to 0.63)
Mongolia	10 (5 to 19)	0.96 (0.45 to 1.87)	32 (20 to 49)	1.89 (1.18 to 2.92)	225.52 (71.13 to 542.21)	2.53 (2.09 to 2.97)
Montenegro	2 (1 to 2)	0.49 (0.31 to 0.7)	1 (1 to 2)	0.36 (0.22 to 0.53)	-33.93 (-56.47 to 0.12)	-1.33 (-1.88 to -0.77)
New Zealand	18 (12 to 24)	1.01 (0.69 to 1.35)	12 (7 to 16)	0.49 (0.31 to 0.67)	-34.81 (-47.38 to -19.17)	-2.28 (-2.5 to -2.07)
North Macedonia	21 (13 to 29)	2 (1.25 to 2.82)	15 (9 to 23)	1.38 (0.84 to 2.05)	-26.39 (-52.97 to 13.35)	-1.26 (-1.66 to -0.86)
Northern Mariana Islands	0 (0 to 1)	1.33 (0.63 to 2.26)	0 (0 to 1)	1.52 (0.92 to 2.27)	-6.76 (-44.92 to 75.74)	0.63 (0.2 to 1.06)
Palestine	1 (1 to 2)	0.12 (0.06 to 0.18)	2 (1 to 3)	0.07 (0.04 to 0.1)	70.07 (1.3 to 184.17)	-1.96 (-2.03 to -1.89)
People's Democratic Republic of Algeria	48 (22 to 88)	0.41 (0.18 to 0.75)	62 (29 to 110)	0.28 (0.13 to 0.49)	30.27 (-33.94 to 142)	-1.67 (-1.9 to -1.44)
People's Republic of Bangladesh	743 (311 to 1472)	1.49 (0.62 to 2.94)	1176 (587 to 2211)	1.34 (0.67 to 2.51)	58.26 (-4.84 to 185.94)	-0.05 (-0.37 to 0.26)
People's Republic of China	6251 (2756 to 10280)	0.94 (0.41 to 1.54)	3124 (1757 to 4755)	0.47 (0.26 to 0.72)	-50.02 (-69.67 to -9.31)	-2.89 (-3.35 to -2.42)
Plurinational State of Bolivia	34 (15 to 64)	1.14 (0.49 to 2.15)	55 (25 to 94)	0.88 (0.39 to 1.51)	60.62 (-17.4 to 254.73)	-1.12 (-1.47 to -0.76)
Portugal	59 (42 to 75)	1.17 (0.85 to 1.49)	26 (17 to 36)	0.57 (0.37 to 0.78)	-55.06 (-65.68 to -43.87)	-1.98 (-2.21 to -1.75)
Principality of Andorra	0 (0 to 0)	0.74 (0.41 to 1.2)	0 (0 to 0)	0.39 (0.19 to 0.64)	-32.06 (-65.51 to 19.47)	-1.79 (-2.01 to -1.56)

Country	Cases_1990	Rates_1990	Cases_2021	Rates_2021	Cases_change	EAPC_CI
Principality of Monaco	0 (0 to 0)	1.35 (0.81 to 2.07)	0 (0 to 0)	0.55 (0.29 to 0.96)	-58.8 (-77.38 to -26.57)	-2.85 (-3 to -2.7)
Puerto Rico	11 (7 to 15)	0.58 (0.35 to 0.84)	6 (4 to 9)	0.42 (0.26 to 0.59)	-43.15 (-61.99 to -16.13)	-1.71 (-1.97 to -1.45)
Republic of Albania	8 (5 to 14)	0.46 (0.28 to 0.79)	5 (3 to 8)	0.39 (0.23 to 0.61)	-37.12 (-64.64 to 8.76)	-0.45 (-0.88 to -0.02)
Republic of Angola	20 (8 to 43)	0.43 (0.16 to 0.92)	50 (19 to 112)	0.34 (0.13 to 0.77)	152.3 (45.38 to 373.45)	-0.81 (-0.94 to -0.67)
Republic of Armenia	9 (6 to 11)	0.51 (0.35 to 0.65)	6 (5 to 8)	0.44 (0.32 to 0.57)	-26.51 (-40.5 to -9.78)	-1.08 (-1.83 to -0.33)
Republic of Austria	36 (25 to 46)	0.89 (0.62 to 1.14)	14 (9 to 19)	0.35 (0.23 to 0.47)	-61.07 (-71.53 to -51.17)	-2.74 (-3.03 to -2.46)
Republic of Azerbaijan	8 (4 to 13)	0.22 (0.11 to 0.34)	8 (4 to 14)	0.15 (0.08 to 0.26)	4.66 (-47.07 to 108.57)	-1.91 (-2.51 to -1.31)
Republic of Belarus	53 (35 to 72)	1.06 (0.69 to 1.43)	59 (41 to 79)	1.38 (0.96 to 1.87)	10.05 (-25.36 to 63.12)	0.45 (0.14 to 0.77)
Republic of Benin	6 (2 to 16)	0.31 (0.1 to 0.81)	20 (7 to 53)	0.32 (0.11 to 0.84)	222.42 (102.98 to 465.38)	0.18 (-0.1 to 0.46)
Republic of Botswana	2 (1 to 4)	0.29 (0.13 to 0.63)	3 (1 to 6)	0.22 (0.11 to 0.47)	71.53 (-4.52 to 234.13)	-1.14 (-1.36 to -0.92)
Republic of Bulgaria	59 (42 to 76)	1.43 (1 to 1.84)	38 (26 to 50)	1.3 (0.88 to 1.71)	-35.53 (-50.93 to -15.58)	-0.35 (-0.57 to -0.12)
Republic of Burundi	18 (5 to 48)	0.73 (0.2 to 1.97)	28 (6 to 97)	0.45 (0.09 to 1.55)	57.34 (-25.44 to 217.11)	-2.28 (-2.61 to -1.95)
Republic of Cabo Verde	1 (0 to 2)	0.44 (0.14 to 1.27)	1 (0 to 3)	0.39 (0.14 to 1.07)	89.84 (19.51 to 211.89)	-0.39 (-0.64 to -0.13)
Republic of Cameroon	13 (4 to 31)	0.29 (0.1 to 0.68)	76 (26 to 175)	0.49 (0.17 to 1.13)	485.23 (237.35 to 943.59)	1.77 (1.06 to 2.49)

Country	Cases_1990	Rates_1990	Cases_2021	Rates_2021	Cases_change	EAPC_CI
Republic of Chad	10 (2 to 31)	0.39 (0.09 to 1.23)	30 (8 to 86)	0.4 (0.11 to 1.16)	206.53 (88.39 to 472.46)	0.04 (-0.22 to 0.3)
Republic of Chile	68 (46 to 90)	0.96 (0.64 to 1.27)	59 (36 to 81)	0.62 (0.37 to 0.85)	-13.36 (-34.68 to 7.65)	-1.31 (-1.65 to -0.98)
Republic of Colombia	135 (79 to 191)	0.8 (0.47 to 1.13)	186 (99 to 275)	0.71 (0.38 to 1.05)	38.11 (-8.38 to 108.14)	-0.01 (-0.2 to 0.19)
Republic of Costa Rica	9 (5 to 12)	0.56 (0.34 to 0.78)	20 (12 to 26)	0.78 (0.49 to 1.05)	126.96 (65.3 to 213.28)	0.48 (0.25 to 0.71)
Republic of Côte d'Ivoire	26 (10 to 60)	0.46 (0.17 to 1.08)	66 (26 to 162)	0.48 (0.19 to 1.18)	154.95 (45.36 to 352.91)	0.13 (-0.03 to 0.3)
Republic of Croatia	34 (23 to 46)	1.4 (0.94 to 1.9)	10 (6 to 12)	0.52 (0.35 to 0.68)	-72.03 (-79.35 to -61.82)	-3.09 (-3.59 to -2.58)
Republic of Cuba	45 (25 to 67)	0.73 (0.41 to 1.09)	24 (13 to 35)	0.46 (0.25 to 0.69)	-47.77 (-68.66 to -13.45)	-1.5 (-1.77 to -1.23)
Republic of Cyprus	4 (2 to 6)	0.98 (0.61 to 1.43)	3 (2 to 4)	0.36 (0.21 to 0.51)	-36.05 (-59.08 to -2.71)	-3.92 (-4.17 to -3.67)
Republic of Djibouti	1 (0 to 2)	0.42 (0.13 to 1.15)	3 (1 to 9)	0.49 (0.15 to 1.27)	287.95 (113.19 to 614.37)	0.46 (0.22 to 0.7)
Republic of Ecuador	26 (12 to 44)	0.52 (0.23 to 0.89)	85 (49 to 128)	0.9 (0.53 to 1.36)	227.57 (100.11 to 549.26)	2.76 (2.39 to 3.13)
Republic of El Salvador	22 (12 to 34)	0.86 (0.48 to 1.34)	21 (12 to 32)	0.63 (0.35 to 0.97)	-3.62 (-40.73 to 57.42)	-0.8 (-1.41 to -0.19)
Republic of Equatorial Guinea	1 (0 to 2)	0.53 (0.2 to 1.22)	2 (1 to 3)	0.2 (0.08 to 0.36)	67.76 (-20.12 to 283.46)	-3.98 (-4.67 to -3.28)
Republic of Estonia	10 (7 to 12)	1.27 (0.88 to 1.59)	3 (2 to 4)	0.46 (0.3 to 0.62)	-72.53 (-80.51 to -63.35)	-4.51 (-5.06 to -3.95)
Republic of Fiji	6 (4 to 9)	1.48 (0.91 to 2.25)	7 (4 to 11)	1.49 (0.91 to 2.27)	19.12 (-19.9 to 88.49)	0.06 (-0.05 to 0.17)

Country	Cases_1990	Rates_1990	Cases_2021	Rates_2021	Cases_change	EAPC_CI
Republic of Finland	67 (50 to 82)	2.59 (1.93 to 3.17)	17 (12 to 21)	0.71 (0.5 to 0.91)	-75.06 (-79.69 to -70.56)	-4.52 (-4.79 to -4.25)
Republic of Ghana	52 (18 to 142)	0.75 (0.27 to 2.07)	105 (41 to 239)	0.6 (0.24 to 1.37)	103.54 (32.55 to 246.49)	-0.74 (-0.98 to -0.5)
Republic of Guatemala	7 (4 to 12)	0.21 (0.1 to 0.34)	47 (31 to 64)	0.56 (0.37 to 0.76)	529.4 (313.18 to 996.78)	3.76 (3.24 to 4.29)
Republic of Guinea	10 (4 to 28)	0.42 (0.14 to 1.1)	28 (10 to 66)	0.46 (0.16 to 1.08)	167.03 (60.28 to 382.29)	0.69 (0.52 to 0.86)
Republic of Guinea-Bissau	3 (1 to 8)	0.76 (0.24 to 1.77)	8 (2 to 18)	0.76 (0.23 to 1.82)	127.11 (28.62 to 298.22)	0.09 (0.05 to 0.13)
Republic of Haiti	75 (36 to 122)	2.55 (1.23 to 4.16)	180 (79 to 301)	2.62 (1.15 to 4.39)	140.62 (40.94 to 321.83)	0.34 (0.22 to 0.46)
Republic of Honduras	29 (15 to 47)	1.41 (0.74 to 2.3)	68 (33 to 118)	1.26 (0.61 to 2.18)	134.92 (25.55 to 330.19)	-0.74 (-0.97 to -0.52)
Republic of Iceland	1 (0 to 1)	0.47 (0.29 to 0.67)	1 (0 to 1)	0.32 (0.19 to 0.44)	-17.27 (-41.23 to 16.49)	-1.59 (-1.79 to -1.4)
Republic of India	3131 (1727 to 5061)	0.74 (0.41 to 1.2)	4453 (2656 to 6818)	0.57 (0.34 to 0.87)	42.22 (12.14 to 87.09)	-0.87 (-1.04 to -0.69)
Republic of Indonesia	1186 (761 to 1793)	1.25 (0.81 to 1.9)	1891 (1223 to 2845)	1.23 (0.8 to 1.86)	59.39 (20.74 to 120.12)	0.27 (0.06 to 0.49)
Republic of Iraq	51 (31 to 78)	0.6 (0.36 to 0.92)	91 (56 to 138)	0.41 (0.25 to 0.62)	78.55 (3.87 to 206.63)	-1.72 (-1.96 to -1.48)
Republic of Italy	187 (131 to 232)	0.65 (0.46 to 0.81)	67 (43 to 90)	0.27 (0.17 to 0.36)	-64.25 (-71.84 to -56.69)	-2.66 (-2.96 to -2.36)
Republic of Kazakhstan	88 (56 to 121)	1.07 (0.68 to 1.47)	93 (64 to 123)	0.99 (0.68 to 1.31)	5.56 (-27.7 to 51.69)	-0.53 (-1.07 to 0.02)
Republic of Kenya	20 (6 to 54)	0.2 (0.06 to 0.54)	67 (25 to 169)	0.26 (0.1 to 0.65)	236.36 (151.95 to 401.98)	1.17 (0.9 to 1.44)
Republic of Kiribati	1 (0 to 1)	1.67 (0.83 to 3.07)	1 (1 to 2)	2.11 (1.05 to 3.63)	114.19 (14.32 to 290.64)	0.5 (0.33 to 0.66)
Republic of	428 (250 to	1.65 (0.97	124 (67 to	0.51 (0.27	-70.94 (-	-4.53 (-

Country	Cases_1990	Rates_1990	Cases_2021	Rates_2021	Cases_change	EAPC_CI
Korea	649)	to 2.51)	195)	to 0.8)	82.77 to -53.41)	4.76 to -4.3)
Republic of Latvia	14 (10 to 19)	1.12 (0.76 to 1.47)	6 (4 to 8)	0.75 (0.5 to 1.02)	-58.85 (-70.48 to -43.56)	-1.95 (-2.5 to -1.4)
Republic of Liberia	5 (1 to 13)	0.43 (0.13 to 1.18)	16 (4 to 57)	0.57 (0.15 to 2.03)	243.2 (47.49 to 564.45)	0.42 (0.16 to 0.68)
Republic of Lithuania	21 (15 to 27)	1.17 (0.83 to 1.46)	10 (7 to 13)	0.89 (0.64 to 1.15)	-51.94 (-63.21 to -38.4)	-0.55 (-1.3 to 0.2)
Republic of Madagascar	44 (13 to 121)	0.83 (0.25 to 2.28)	123 (34 to 386)	0.87 (0.24 to 2.73)	181.26 (60.87 to 369.94)	0.26 (0.17 to 0.36)
Republic of Malawi	20 (6 to 53)	0.45 (0.13 to 1.2)	47 (12 to 146)	0.48 (0.12 to 1.5)	133.13 (43.61 to 252.98)	-0.03 (-0.31 to 0.24)
Republic of Maldives	1 (0 to 1)	0.84 (0.48 to 1.32)	1 (1 to 2)	0.34 (0.19 to 0.54)	41.65 (-14.48 to 132.55)	-3.28 (-3.65 to -2.91)
Republic of Mali	13 (4 to 35)	0.37 (0.11 to 0.96)	28 (10 to 67)	0.27 (0.1 to 0.64)	109.96 (19.55 to 298.6)	-0.96 (-1.17 to -0.75)
Republic of Malta	1 (1 to 1)	0.5 (0.35 to 0.64)	1 (0 to 1)	0.31 (0.19 to 0.42)	-35.91 (-53.41 to -18.71)	-1.63 (-1.79 to -1.47)
Republic of Mauritius	7 (5 to 10)	1.23 (0.82 to 1.63)	10 (7 to 13)	1.56 (1.05 to 2.04)	34.36 (5.44 to 72)	1.63 (1.14 to 2.12)
Republic of Moldova	11 (7 to 15)	0.51 (0.34 to 0.68)	9 (6 to 12)	0.49 (0.34 to 0.65)	-21.63 (-44.24 to 8.47)	-0.75 (-1.49 to -0.01)
Republic of Mozambique	39 (11 to 117)	0.68 (0.2 to 2.02)	129 (35 to 388)	0.9 (0.24 to 2.72)	229.06 (101.41 to 447.41)	1.67 (1.37 to 1.98)
Republic of Namibia	1 (1 to 3)	0.23 (0.11 to 0.44)	3 (2 to 7)	0.25 (0.12 to 0.5)	120.75 (22.76 to 286.27)	0.02 (-0.37 to 0.4)
Republic of Nauru	0 (0 to 0)	3.24 (1.41 to 5.4)	0 (0 to 0)	3.53 (2.1 to 5.33)	26 (-25.14 to 126.4)	-0.03 (-0.68 to 0.62)
Republic of Nicaragua	11 (7 to 16)	0.64 (0.38 to 0.95)	20 (12 to 29)	0.55 (0.34 to 0.8)	78.46 (13.59 to 179.12)	-0.24 (-0.42 to -

Country	Cases_1990	Rates_1990	Cases_2021	Rates_2021	Cases_change	EAPC_CI
						0.05)
Republic of Niue	0 (0 to 0)	1.78 (0.94 to 3.13)	0 (0 to 0)	1.59 (0.93 to 2.56)	-31.76 (-63.8 to 24.84)	-0.87 (-1.09 to -0.66)
Republic of Palau	0 (0 to 0)	1.42 (0.67 to 2.43)	0 (0 to 0)	2.32 (1.3 to 3.5)	73.07 (-0.26 to 239.69)	1.49 (1.25 to 1.72)
Republic of Panama	8 (5 to 11)	0.63 (0.39 to 0.88)	17 (10 to 25)	0.8 (0.48 to 1.15)	124.38 (56.07 to 245.34)	1.37 (1.08 to 1.65)
Republic of Paraguay	27 (17 to 38)	1.43 (0.91 to 2.02)	39 (22 to 61)	1 (0.57 to 1.57)	42.5 (-14.41 to 126.35)	-1.12 (-1.23 to -1)
Republic of Peru	53 (21 to 90)	0.5 (0.2 to 0.84)	138 (74 to 214)	0.71 (0.38 to 1.11)	158.87 (37.41 to 406.39)	1.71 (1.25 to 2.18)
Republic of Poland	329 (229 to 416)	1.73 (1.2 to 2.2)	154 (107 to 197)	0.86 (0.6 to 1.1)	-53.05 (-61.32 to -44.09)	-2.55 (-2.82 to -2.28)
Republic of Rwanda	23 (7 to 55)	0.72 (0.21 to 1.73)	21 (5 to 69)	0.31 (0.08 to 1.01)	-6.68 (-49.82 to 62.87)	-4.19 (-4.78 to -3.59)
Republic of San Marino	0 (0 to 0)	0.37 (0.23 to 0.53)	0 (0 to 0)	0.17 (0.08 to 0.29)	-50.19 (-73.3 to -12.42)	-1.71 (-2 to -1.41)
Republic of Senegal	17 (6 to 39)	0.51 (0.19 to 1.21)	32 (11 to 85)	0.41 (0.14 to 1.1)	91.7 (20.02 to 206.81)	-0.48 (-0.73 to -0.23)
Republic of Serbia	108 (71 to 149)	2.27 (1.5 to 3.12)	46 (31 to 63)	1.08 (0.72 to 1.48)	-57.9 (-71.26 to -38.05)	-2.97 (-3.36 to -2.57)
Republic of Seychelles	0 (0 to 0)	0.84 (0.48 to 1.26)	0 (0 to 0)	0.53 (0.28 to 0.86)	-7.29 (-37.79 to 27.16)	-1.07 (-1.23 to -0.91)
Republic of Sierra Leone	10 (4 to 24)	0.52 (0.18 to 1.28)	23 (8 to 65)	0.53 (0.17 to 1.45)	138.49 (38.76 to 292.56)	0.16 (-0.07 to 0.38)
Republic of Singapore	10 (6 to 14)	0.55 (0.34 to 0.76)	5 (3 to 8)	0.18 (0.09 to 0.27)	-49.65 (-67.76 to -28.67)	-4.61 (-4.98 to -4.22)
Republic of Slovenia	8 (6 to 11)	0.8 (0.54 to 1.07)	2 (1 to 3)	0.24 (0.15 to 0.33)	-74.44 (-82.07 to -64.33)	-4.25 (-4.76 to -3.74)
Republic of	63 (42 to	0.34 (0.22	92 (61 to	0.29 (0.2 to	44.59 (8.27 to	-0.42 (-

Country	Cases_1990	Rates_1990	Cases_2021	Rates_2021	Cases_change	EAPC_CI
South Africa	88)	to 0.46)	129)	0.41)	89.09)	0.81 to -0.03)
Republic of South Sudan	8 (3 to 20)	0.31 (0.12 to 0.73)	16 (5 to 41)	0.37 (0.12 to 0.92)	92.5 (-4.58 to 275.19)	0.48 (0.02 to 0.95)
Republic of Sudan	79 (29 to 137)	0.87 (0.32 to 1.5)	108 (43 to 209)	0.48 (0.19 to 0.94)	36.51 (-24.95 to 141.49)	-2.03 (-2.11 to -1.96)
Republic of Suriname	1 (1 to 2)	0.73 (0.38 to 1.1)	2 (1 to 4)	0.79 (0.43 to 1.24)	57.22 (-9.09 to 171.31)	0.07 (-0.13 to 0.27)
Republic of Tajikistan	13 (7 to 20)	0.52 (0.28 to 0.85)	24 (13 to 43)	0.47 (0.25 to 0.84)	92.02 (-0.99 to 274.37)	-1.01 (-1.32 to -0.71)
Republic of the Congo	5 (2 to 10)	0.47 (0.21 to 0.93)	13 (6 to 27)	0.45 (0.2 to 0.97)	146.36 (39.64 to 363.63)	-0.36 (-0.55 to -0.17)
Republic of the Gambia	2 (1 to 5)	0.42 (0.15 to 1.05)	6 (2 to 17)	0.52 (0.17 to 1.42)	231.68 (77.95 to 422.92)	0.49 (0.22 to 0.76)
Republic of the Marshall Islands	0 (0 to 1)	1.65 (0.76 to 3.02)	1 (0 to 1)	2.29 (1.07 to 4.02)	110.64 (15.91 to 264.7)	0.91 (0.81 to 1.01)
Republic of the Niger	13 (3 to 34)	0.37 (0.1 to 1)	29 (9 to 85)	0.28 (0.08 to 0.82)	131.13 (34.35 to 315.35)	-0.67 (-0.83 to -0.51)
Republic of the Philippines	163 (96 to 233)	0.52 (0.31 to 0.75)	431 (259 to 615)	0.72 (0.43 to 1.02)	163.98 (89.74 to 259.62)	1.58 (1.37 to 1.78)
Republic of the Union of Myanmar	268 (133 to 457)	1.31 (0.65 to 2.24)	310 (180 to 497)	1.05 (0.61 to 1.69)	15.61 (-31.17 to 106.13)	-0.57 (-0.88 to -0.26)
Republic of Trinidad and Tobago	5 (3 to 7)	0.77 (0.44 to 1.11)	11 (7 to 16)	1.57 (0.99 to 2.31)	127.64 (50.22 to 254.32)	2.04 (1.81 to 2.28)
Republic of Tunisia	12 (6 to 23)	0.3 (0.14 to 0.56)	16 (7 to 30)	0.27 (0.11 to 0.49)	30.9 (-35.63 to 154.89)	-0.7 (-0.79 to -0.6)
Republic of Turkey	350 (193 to 564)	1.21 (0.67 to 1.94)	225 (129 to 326)	0.51 (0.29 to 0.74)	-35.6 (-61.12 to 11.31)	-3.11 (-3.24 to -2.97)
Republic of Uganda	28 (7 to 76)	0.38 (0.09 to 1.03)	53 (14 to 145)	0.26 (0.07 to 0.72)	90.57 (13.95 to 244.92)	-2.01 (-2.33 to -

Country	Cases_1990	Rates_1990	Cases_2021	Rates_2021	Cases_change	EAPC_CI
						1.69)
Republic of Uzbekistan	24 (14 to 35)	0.25 (0.15 to 0.36)	89 (61 to 119)	0.5 (0.34 to 0.67)	271.54 (161.55 to 446.59)	2.48 (2.02 to 2.95)
Republic of Vanuatu	2 (1 to 3)	2.55 (1.39 to 4.29)	4 (2 to 7)	2.56 (1.35 to 4.42)	123.45 (27.08 to 269.37)	-0.3 (-0.4 to -0.2)
Republic of Yemen	33 (13 to 64)	0.6 (0.24 to 1.18)	78 (31 to 157)	0.46 (0.19 to 0.94)	137.61 (26.64 to 354.22)	-1.28 (-1.57 to -1)
Republic of Zambia	10 (3 to 28)	0.28 (0.08 to 0.79)	43 (13 to 105)	0.44 (0.14 to 1.09)	329.26 (122.53 to 794.92)	1.66 (1.55 to 1.78)
Republic of Zimbabwe	11 (6 to 20)	0.25 (0.13 to 0.43)	60 (30 to 107)	0.77 (0.38 to 1.39)	426.27 (216.59 to 744.54)	4.71 (3.8 to 5.63)
Romania	132 (91 to 172)	1.16 (0.8 to 1.51)	97 (67 to 127)	1.16 (0.8 to 1.53)	-26.41 (-44.14 to -2.87)	-0.37 (-0.91 to 0.18)
Russian Federation	951 (695 to 1166)	1.28 (0.94 to 1.57)	1111 (785 to 1388)	1.65 (1.16 to 2.06)	16.85 (5.68 to 27.52)	-0.36 (-1.09 to 0.38)
Saint Kitts and Nevis	0 (0 to 0)	1.3 (0.79 to 1.89)	0 (0 to 0)	0.91 (0.58 to 1.32)	10.41 (-28.18 to 79.1)	-1.98 (-2.37 to -1.59)
Saint Lucia	1 (0 to 1)	0.95 (0.61 to 1.29)	1 (0 to 1)	0.84 (0.5 to 1.19)	22.35 (-10.36 to 64.95)	-0.26 (-0.52 to 0)
Saint Vincent and the Grenadines	0 (0 to 0)	0.51 (0.3 to 0.75)	0 (0 to 1)	0.77 (0.47 to 1.08)	59.21 (9.1 to 143.63)	1.21 (0.86 to 1.57)
Slovak Republic	29 (19 to 41)	1.08 (0.72 to 1.53)	15 (10 to 21)	0.58 (0.38 to 0.82)	-48.12 (-64.77 to -21.83)	-1.86 (-2.02 to -1.69)
Socialist Republic of Viet Nam	223 (110 to 454)	0.68 (0.34 to 1.38)	441 (245 to 747)	0.85 (0.47 to 1.43)	97.8 (8.02 to 281.81)	1.31 (1.06 to 1.56)
Solomon Islands	1 (1 to 3)	0.97 (0.35 to 1.99)	4 (2 to 8)	1.3 (0.58 to 2.4)	205.66 (47.03 to 629.42)	1.2 (1.03 to 1.37)
State of Eritrea	10 (2 to 27)	0.63 (0.16 to 1.74)	18 (4 to 48)	0.52 (0.13 to 1.4)	83.53 (-1.86 to 233.58)	-0.46 (-0.7 to -0.21)

Country	Cases_1990	Rates_1990	Cases_2021	Rates_2021	Cases_change	EAPC_CI
State of Israel	9 (6 to 12)	0.39 (0.26 to 0.51)	7 (4 to 9)	0.15 (0.1 to 0.2)	-28.35 (-45.21 to -6.22)	-3.05 (-3.28 to -2.83)
State of Kuwait	2 (1 to 3)	0.2 (0.12 to 0.28)	2 (2 to 4)	0.08 (0.05 to 0.12)	18.98 (-15.67 to 69.23)	-3.67 (-4.35 to -2.99)
State of Libya	6 (3 to 9)	0.28 (0.15 to 0.47)	19 (8 to 33)	0.46 (0.19 to 0.8)	236.5 (64.79 to 469.06)	1.94 (1.66 to 2.21)
State of Qatar	2 (1 to 3)	0.61 (0.33 to 1.1)	6 (3 to 10)	0.27 (0.15 to 0.44)	237.84 (68.03 to 551.29)	-3.46 (-3.98 to -2.93)
Sultanate of Oman	3 (1 to 5)	0.26 (0.12 to 0.48)	5 (3 to 8)	0.17 (0.09 to 0.28)	96.28 (6.93 to 273.97)	-1.16 (-1.35 to -0.96)
Swiss Confederation	24 (16 to 32)	0.66 (0.44 to 0.88)	6 (4 to 9)	0.15 (0.09 to 0.21)	-74.78 (-82.36 to -66.92)	-4.9 (-5.22 to -4.59)
Syrian Arab Republic	24 (12 to 41)	0.44 (0.22 to 0.72)	23 (13 to 38)	0.33 (0.18 to 0.53)	-5.09 (-58.3 to 98.15)	-0.96 (-1.59 to -0.32)
Taiwan (Province of China)	36 (22 to 49)	0.32 (0.2 to 0.44)	44 (24 to 63)	0.39 (0.21 to 0.55)	24.76 (-15.82 to 74.74)	-0.06 (-0.41 to 0.3)
Togolese Republic	8 (3 to 21)	0.5 (0.17 to 1.28)	22 (7 to 59)	0.52 (0.17 to 1.41)	171.92 (59.03 to 347.4)	0.17 (-0.1 to 0.44)
Tokelau	0 (0 to 0)	1.46 (0.61 to 2.83)	0 (0 to 0)	1.67 (0.94 to 2.92)	7.88 (-41.16 to 113.05)	-0.12 (-0.33 to 0.1)
Turkmenistan	14 (9 to 21)	0.82 (0.52 to 1.17)	46 (29 to 71)	1.73 (1.07 to 2.66)	219.46 (117.19 to 378.48)	2.59 (2.1 to 3.07)
Tuvalu	0 (0 to 0)	2.18 (0.89 to 4.23)	0 (0 to 0)	2.27 (1.18 to 3.84)	43.84 (-17.62 to 165.62)	-0.11 (-0.34 to 0.12)
Ukraine	247 (170 to 323)	0.99 (0.68 to 1.3)	296 (183 to 434)	1.46 (0.9 to 2.15)	19.67 (-20.17 to 83.98)	1.08 (0.9 to 1.25)
Union of the Comoros	1 (0 to 2)	0.48 (0.18 to 1.08)	2 (1 to 4)	0.42 (0.15 to 1.14)	68.88 (-9.25 to 210.24)	-0.75 (-1.2 to -0.29)
United Arab	7 (3 to 11)	0.55 (0.29	21 (12 to	0.3 (0.17 to	215.95 (87.21	-2.35 (-

Country	Cases_1990	Rates_1990	Cases_2021	Rates_2021	Cases_change	EAPC_CI
Emirates		to 0.9)	33)	0.48)	to 533.14)	2.82 to - 1.88)
United Kingdom of Great Britain and Northern Ireland	409 (304 to 501)	1.44 (1.07 to 1.76)	141 (101 to 178)	0.46 (0.33 to 0.59)	-65.6 (-68.91 to -62.74)	-4.17 (- 4.46 to - 3.88)
United Mexican States	186 (115 to 267)	0.44 (0.27 to 0.63)	469 (308 to 631)	0.69 (0.45 to 0.92)	152.54 (79.58 to 268.68)	1.53 (1.44 to 1.61)
United Republic of Tanzania	40 (11 to 116)	0.35 (0.1 to 1.03)	90 (28 to 284)	0.32 (0.1 to 1.01)	125.96 (43.1 to 269.78)	-0.56 (- 0.74 to - 0.38)
United States of America	922 (590 to 1244)	0.69 (0.44 to 0.93)	700 (427 to 949)	0.46 (0.28 to 0.62)	-24.05 (- 37.67 to - 8.22)	-1.71 (- 2.04 to - 1.38)
United States Virgin Islands	1 (0 to 1)	1.14 (0.62 to 1.89)	0 (0 to 0)	0.77 (0.38 to 1.21)	-58.34 (- 77.96 to - 30.34)	-0.68 (- 0.87 to - 0.49)

Supplementary Table S3. Trends in subarachnoid hemorrhage DALYs attributable to high systolic blood pressure in 204 countries from 1990 to 2021.

Country	Cases_1990	Rates_1990	Cases_2021	Rates_2021	Cases_change	EAPC_CI
American Samoa	17 (11 to 24)	70.22 (43.21 to 97.92)	21 (13 to 31)	88.88 (54.84 to 130.81)	24.65 (-21.33 to 89.32)	0.59 (0.43 to 0.75)
Antigua and Barbuda	13 (7 to 20)	42.46 (23.75 to 62.58)	15 (9 to 20)	30.83 (18.15 to 43.11)	9.5 (-28.38 to 65.62)	-0.26 (-0.52 to 0.01)
Arab Republic of Egypt	7134 (3277 to 12556)	26.58 (12.21 to 46.78)	10200 (6104 to 16427)	19.08 (11.42 to 30.73)	42.98 (-15.66 to 165.72)	-0.8 (-0.95 to -0.65)
Argentine Republic	11683 (6420 to 18176)	73.51 (40.39 to 114.36)	9693 (6111 to 13382)	41.18 (25.96 to 56.85)	-17.03 (-41.64 to 25.26)	-1.65 (-1.84 to -1.45)
Australia	3308 (2178 to 4436)	36.81 (24.23 to 49.36)	2549 (1549 to 3618)	21.23 (12.9 to 30.13)	-22.94 (-40.39 to -4.14)	-2.24 (-2.51 to -1.97)
Barbados	65 (40 to 91)	48.12 (29.87 to 67.47)	74 (45 to 105)	52.91 (32.1 to 75.46)	14.09 (-19.83 to 63.06)	0.44 (0.27 to 0.62)
Belize	21 (11 to 32)	24.46 (13.29 to 37.67)	57 (32 to 81)	23.87 (13.61 to 34.14)	171.86 (77.67 to 347.98)	-0.09 (-0.3 to 0.12)
Bermuda	11 (6 to 17)	32.49 (18.08 to 49.12)	6 (3 to 8)	20.64 (11.46 to 30.43)	-49.57 (-68.28 to -19.46)	-1.64 (-1.84 to -1.43)
Bolivarian Republic of Venezuela	6266 (4275 to 8171)	65.34 (44.58 to 85.21)	7766 (4327 to 11997)	59.07 (32.91 to 91.24)	23.95 (-19.65 to 74.34)	-1.28 (-1.8 to -0.75)
Bosnia and Herzegovina	1952 (1235 to 2775)	81.53 (51.61 to 115.92)	639 (390 to 902)	42.91 (26.2 to 60.55)	-67.26 (-79.06 to -50.09)	-2.69 (-3 to -2.37)
Brunei Darussalam	147 (86 to 215)	100.18 (58.87 to 146.77)	204 (128 to 289)	75.23 (47.26 to 106.57)	39.21 (-6.76 to 115.72)	-1.22 (-1.61 to -0.83)
Burkina Faso	658 (221 to 1704)	17.16 (5.75 to 44.44)	1517 (511 to 3600)	14.72 (4.96 to 34.92)	130.63 (52.66 to 286.48)	-0.48 (-0.63 to -0.33)
Canada	5235 (3350 to 7310)	35.5 (22.72 to 49.57)	2995 (1626 to 4588)	18.01 (9.78 to 27.58)	-42.78 (-59.94 to -23.33)	-2.78 (-3.1 to -2.45)

Country	Cases_1990	Rates_1990	Cases_2021	Rates_2021	Cases_change	EAPC_CI
Central African Republic	387 (154 to 884)	31.17 (12.37 to 71.16)	822 (273 to 1986)	30.83 (10.25 to 74.49)	112.37 (22.19 to 259.15)	-0.1 (-0.17 to -0.03)
Commonwealth of Dominica	12 (7 to 18)	33.47 (19.43 to 50.94)	13 (8 to 20)	39.13 (23.73 to 59.33)	11.69 (-28.15 to 71.03)	0.62 (0.48 to 0.75)
Commonwealth of the Bahamas	55 (32 to 78)	38.7 (22.75 to 54.9)	94 (57 to 138)	44.96 (27.41 to 65.61)	71.11 (6.52 to 181.04)	0.58 (0.38 to 0.77)
Cook Islands	6 (3 to 10)	58.59 (27.9 to 102.92)	4 (2 to 6)	44.74 (28.21 to 69.51)	-35.16 (-63.38 to 26.12)	-0.62 (-0.78 to -0.46)
Czech Republic	4157 (3038 to 5258)	79.84 (58.34 to 100.98)	1532 (1000 to 2083)	32.47 (21.21 to 44.15)	-63.16 (-71.4 to -53.89)	-2.32 (-2.6 to -2.04)
Democratic People's Republic of Korea	6432 (3552 to 10888)	59.86 (33.06 to 101.34)	6393 (2967 to 12761)	46.23 (21.45 to 92.27)	-0.6 (-47.71 to 91.89)	-1.26 (-1.45 to -1.06)
Democratic Republic of Sao Tome and Principe	12 (5 to 30)	24.33 (10.11 to 60.25)	38 (15 to 103)	34.04 (13.57 to 91.18)	215.75 (88.5 to 409.01)	1.1 (0.73 to 1.47)
Democratic Republic of the Congo	3425 (1405 to 7705)	20.4 (8.37 to 45.9)	7985 (2585 to 26192)	18.43 (5.97 to 60.47)	133.14 (27.05 to 327.67)	-0.49 (-0.68 to -0.31)
Democratic Republic of Timor-Leste	185 (99 to 321)	48.21 (25.74 to 83.74)	319 (182 to 594)	46.25 (26.35 to 86.15)	72.8 (5.64 to 190.97)	-0.06 (-0.4 to 0.28)
Democratic Socialist Republic of Sri Lanka	2461 (1452 to 3739)	26.79 (15.81 to 40.71)	2435 (1489 to 3539)	22.08 (13.51 to 32.1)	-1.07 (-35.68 to 53.92)	-0.44 (-0.62 to -0.26)
Dominican Republic	1350 (691 to 2169)	37.12 (19.02 to 59.65)	2548 (1414 to 3945)	43.47 (24.12 to 67.3)	88.82 (12.46 to 230.89)	1.08 (0.86 to 1.29)
Eastern Republic of Uruguay	1924 (1166 to 2733)	129.48 (78.45 to 183.98)	1143 (721 to 1539)	69.67 (43.91 to 93.77)	-40.57 (-56.98 to -14.45)	-2.25 (-2.47 to -2.02)
Federal Democratic Republic of	3063 (759 to 8376)	14.11 (3.5 to 38.58)	6549 (2286 to 18510)	11.9 (4.15 to 33.63)	113.82 (42.29 to 257.19)	-0.56 (-0.89 to -0.22)

Country	Cases_1990	Rates_1990	Cases_2021	Rates_2021	Cases_change	EAPC_CI
Ethiopia						
Federal Democratic Republic of Nepal	3962 (1988 to 7166)	44.51 (22.33 to 80.5)	4123 (1920 to 7550)	24.77 (11.54 to 45.35)	4.09 (-40.77 to 70.38)	-1.91 (-2.04 to -1.77)
Federal Republic of Germany	25875 (18116 to 32957)	64.87 (45.42 to 82.63)	7909 (5172 to 10599)	22.23 (14.54 to 29.79)	-69.44 (-76.53 to -60.99)	-3.65 (-3.85 to -3.45)
Federal Republic of Nigeria	5818 (2181 to 15534)	14.18 (5.32 to 37.87)	14152 (6595 to 35681)	13.12 (6.11 to 33.08)	143.26 (52.19 to 286.86)	0.02 (-0.2 to 0.24)
Federal Republic of Somalia	1159 (262 to 3728)	33.17 (7.51 to 106.7)	2708 (542 to 9816)	27.18 (5.44 to 98.53)	133.67 (23.33 to 333.99)	-1.03 (-1.31 to -0.75)
Federated States of Micronesia	50 (24 to 87)	106.62 (50.55 to 182.99)	61 (30 to 103)	113.05 (56.34 to 190.84)	20.62 (-28.53 to 108.29)	0.14 (0.08 to 0.2)
Federative Republic of Brazil	74906 (50336 to 98312)	97.75 (65.69 to 128.3)	74951 (51847 to 97036)	64.63 (44.71 to 83.67)	0.06 (-9.89 to 11.7)	-1.72 (-1.91 to -1.52)
French Republic	9567 (6551 to 12449)	32.79 (22.45 to 42.66)	6826 (4545 to 9348)	24.13 (16.07 to 33.05)	-28.65 (-45.53 to -11.48)	-0.9 (-1.25 to -0.55)
Gabonese Republic	74 (36 to 138)	16.49 (8.16 to 30.9)	151 (76 to 273)	16.33 (8.21 to 29.38)	105.34 (22.52 to 261.97)	-0.25 (-0.48 to -0.01)
Georgia	1825 (1230 to 2437)	68.04 (45.85 to 90.86)	1519 (1027 to 2076)	94.92 (64.2 to 129.75)	-16.78 (-41.6 to 19.24)	1.27 (0.74 to 1.79)
Grand Duchy of Luxembourg	90 (61 to 121)	45.38 (30.5 to 61.03)	45 (29 to 61)	14.29 (9.25 to 19.2)	-49.77 (-62.82 to -31.09)	-3.96 (-4.26 to -3.65)
Greenland	49 (27 to 79)	147.44 (79.72 to 235.12)	14 (7 to 23)	54.04 (24.93 to 87.42)	-71.01 (-84.18 to -50.07)	-3.55 (-3.88 to -3.21)
Grenada	20 (11 to 30)	51.5 (27.82 to 76.5)	23 (14 to 33)	43.53 (26.55 to 62.38)	15.29 (-25.28 to 90)	-0.34 (-0.58 to -0.1)
Guam	28 (14 to 44)	36.3 (18.65 to 56.47)	46 (29 to 64)	61.56 (39.19 to 85.94)	63.77 (10.57 to 203.1)	1.46 (1.19 to 1.72)

Country	Cases_1990	Rates_1990	Cases_2021	Rates_2021	Cases_change	EAPC_CI
Guyana	201 (115 to 304)	49.82 (28.53 to 75.5)	245 (144 to 372)	61.17 (36.07 to 93.04)	21.83 (-19.77 to 92.49)	0.77 (0.42 to 1.11)
Hashemite Kingdom of Jordan	143 (82 to 222)	8.02 (4.6 to 12.48)	433 (264 to 641)	6.33 (3.86 to 9.37)	202.93 (90.56 to 388.12)	-1 (-1.11 to -0.89)
Hellenic Republic	1434 (916 to 1990)	28.39 (18.13 to 39.4)	1149 (651 to 1642)	26.55 (15.05 to 37.93)	-19.85 (-39.3 to 5.45)	-0.26 (-0.42 to -0.1)
Hungary	5112 (3703 to 6365)	100.43 (72.76 to 125.07)	1783 (1199 to 2390)	41.08 (27.62 to 55.05)	-65.11 (-73.34 to -55.86)	-3.23 (-3.41 to -3.04)
Independent State of Papua New Guinea	677 (277 to 1355)	34.04 (13.91 to 68.16)	2777 (1245 to 5095)	52 (23.31 to 95.41)	310.38 (134.17 to 734.15)	1.53 (1.18 to 1.89)
Independent State of Samoa	51 (26 to 88)	65.02 (32.68 to 111.91)	89 (49 to 148)	88.46 (49.08 to 147.56)	74.18 (3.6 to 183.88)	0.9 (0.7 to 1.1)
Ireland	1116 (759 to 1409)	62.63 (42.63 to 79.07)	593 (390 to 779)	25.68 (16.91 to 33.75)	-46.87 (-59.91 to -31.44)	-2.99 (-3.3 to -2.68)
Islamic Republic of Afghanistan	1852 (729 to 3560)	46.31 (18.23 to 89.01)	4910 (2040 to 9695)	33.22 (13.8 to 65.6)	165.12 (44.7 to 383.58)	-0.32 (-0.64 to 0.01)
Islamic Republic of Iran	2899 (1715 to 4382)	11.35 (6.72 to 17.16)	4726 (2990 to 6995)	9.97 (6.31 to 14.76)	63.03 (7.81 to 115.34)	-0.33 (-0.64 to -0.03)
Islamic Republic of Mauritania	251 (102 to 617)	27.49 (11.2 to 67.71)	373 (147 to 1033)	18.14 (7.16 to 50.23)	48.85 (-8.69 to 140.31)	-1.41 (-1.51 to -1.32)
Islamic Republic of Pakistan	17230 (9732 to 28151)	34.85 (19.68 to 56.93)	59011 (34347 to 93858)	48.37 (28.16 to 76.94)	242.49 (140.43 to 385.97)	0.92 (0.8 to 1.04)
Jamaica	410 (223 to 629)	35.24 (19.18 to 54)	655 (372 to 958)	42.78 (24.31 to 62.56)	59.6 (8.22 to 156.58)	0.99 (0.66 to 1.32)
Japan	63794 (45806 to 78452)	98.27 (70.56 to 120.85)	28866 (18942 to 38226)	56.96 (37.38 to 75.43)	-54.75 (-63.77 to -45.42)	-2 (-2.29 to -1.71)
Kingdom of Bahrain	57 (35 to 88)	19.04 (11.45 to	137 (87 to 204)	13.99 (8.84 to 20.81)	138.47 (51.4 to 256.56)	-1.66 (-1.95 to -

Country	Cases_1990	Rates_1990	Cases_2021	Rates_2021	Cases_change	EAPC_CI
		29.26)				1.37)
Kingdom of Belgium	1577 (1006 to 2130)	31.8 (20.29 to 42.95)	1320 (912 to 1754)	26.43 (18.26 to 35.13)	-16.32 (-32.41 to 6.54)	-0.68 (-1.15 to -0.21)
Kingdom of Bhutan	89 (41 to 170)	28.49 (13.27 to 54.44)	109 (57 to 182)	25.02 (12.95 to 41.61)	23.19 (-28.66 to 113.83)	-0.93 (-1.09 to -0.78)
Kingdom of Cambodia	1695 (798 to 2974)	36.89 (17.38 to 64.74)	2092 (991 to 3835)	23.21 (10.99 to 42.54)	23.47 (-32.04 to 122.27)	-1.94 (-2.16 to -1.72)
Kingdom of Denmark	1797 (1240 to 2285)	67.24 (46.4 to 85.49)	559 (354 to 768)	21.74 (13.77 to 29.86)	-68.89 (-76.7 to -59.95)	-4.01 (-4.48 to -3.54)
Kingdom of Eswatini	46 (24 to 87)	13.03 (6.61 to 24.56)	115 (62 to 214)	18.73 (10 to 34.74)	148.87 (41.94 to 333.55)	1.49 (0.96 to 2.03)
Kingdom of Lesotho	81 (33 to 172)	12.21 (4.91 to 25.99)	237 (116 to 452)	23.57 (11.53 to 44.97)	192.18 (60.81 to 420.62)	2.68 (2.26 to 3.1)
Kingdom of Morocco	4982 (2289 to 8756)	40.03 (18.39 to 70.36)	5542 (2853 to 9876)	28.53 (14.69 to 50.84)	11.24 (-28.23 to 85.87)	-1.31 (-1.45 to -1.16)
Kingdom of Norway	1510 (1087 to 1880)	69.7 (50.17 to 86.76)	492 (352 to 642)	19.72 (14.08 to 25.71)	-67.4 (-73.35 to -61.54)	-4.19 (-4.42 to -3.95)
Kingdom of Saudi Arabia	1330 (785 to 2118)	16.58 (9.78 to 26.4)	4132 (2116 to 6967)	16.33 (8.36 to 27.54)	210.73 (53.16 to 455.36)	-0.18 (-0.32 to -0.03)
Kingdom of Spain	4487 (2825 to 6211)	23.1 (14.55 to 31.98)	4361 (2900 to 5884)	21.82 (14.51 to 29.43)	-2.79 (-26.45 to 31.91)	-0.54 (-0.89 to -0.19)
Kingdom of Sweden	1910 (1290 to 2513)	45.46 (30.71 to 59.81)	447 (262 to 664)	9.88 (5.79 to 14.68)	-76.58 (-83 to -69.3)	-4.98 (-5.17 to -4.78)
Kingdom of Thailand	15642 (8517 to 26258)	49.41 (26.9 to 82.95)	25513 (14758 to 40926)	79.93 (46.24 to 128.22)	63.11 (-4.9 to 167.78)	1.43 (1.03 to 1.83)
Kingdom of the Netherlands	3577 (2269 to 4866)	44.13 (28 to 60.02)	1340 (872 to 1868)	18.09 (11.78 to 25.23)	-62.55 (-72.26 to -49.29)	-3.05 (-3.51 to -2.6)
Kingdom of	16 (9 to 27)	36.56 (20.24 to	22 (13 to	44.69 (27	35.8 (-8.85 to	0.91 (0.78

Country	Cases_1990	Rates_1990	Cases_2021	Rates_2021	Cases_change	EAPC_CI
Tonga		60.38)	36)	to 73.52)	110.45)	to 1.05)
Kyrgyz Republic	989 (539 to 1440)	47.21 (25.74 to 68.72)	1613 (938 to 2402)	46.92 (27.28 to 69.86)	63.1 (1.15 to 161.76)	-0.93 (-1.48 to -0.39)
Lao People's Democratic Republic	1591 (799 to 2751)	85.59 (42.98 to 148.02)	1940 (1117 to 3084)	48.48 (27.91 to 77.06)	21.98 (-28.1 to 121.1)	-2.05 (-2.14 to -1.96)
Lebanon	729 (397 to 1161)	50.67 (27.61 to 80.74)	664 (446 to 917)	22.04 (14.79 to 30.43)	-8.81 (-44.75 to 53.51)	-2.76 (-3.37 to -2.15)
Malaysia	3351 (2125 to 4868)	37.19 (23.58 to 54.04)	6406 (4373 to 8500)	36.09 (24.64 to 47.89)	91.19 (33.52 to 176.93)	0.39 (0.05 to 0.73)
Mongolia	526 (263 to 981)	51.17 (25.62 to 95.51)	1638 (1027 to 2486)	97.14 (60.89 to 147.37)	211.77 (73.29 to 479.08)	2.32 (1.92 to 2.73)
Montenegro	93 (61 to 130)	29.3 (19.15 to 40.69)	66 (42 to 95)	22.71 (14.46 to 32.47)	-29.02 (-49.74 to 2.95)	-1.09 (-1.54 to -0.64)
New Zealand	931 (634 to 1242)	51.61 (35.11 to 68.85)	630 (405 to 848)	25.91 (16.65 to 34.85)	-32.33 (-45.19 to -16.6)	-2.22 (-2.42 to -2.02)
North Macedonia	1106 (692 to 1526)	107.09 (67.01 to 147.75)	837 (528 to 1198)	76.03 (47.95 to 108.86)	-24.32 (-49.45 to 12.71)	-1.17 (-1.52 to -0.82)
Northern Mariana Islands	22 (10 to 36)	74.18 (35.42 to 124.62)	20 (12 to 30)	85.24 (52.51 to 125.05)	-6.12 (-42.42 to 67.1)	0.53 (0.14 to 0.92)
Palestine	73 (43 to 109)	8.21 (4.84 to 12.32)	159 (92 to 238)	5.97 (3.45 to 8.96)	117.72 (41.43 to 218.93)	-1.18 (-1.3 to -1.06)
People's Democratic Republic of Algeria	2795 (1356 to 4938)	23.74 (11.52 to 41.95)	3868 (2001 to 6537)	17.08 (8.84 to 28.87)	38.41 (-20.07 to 140.65)	-1.47 (-1.7 to -1.24)
People's Republic of Bangladesh	39496 (16697 to 77205)	79.03 (33.41 to 154.48)	64120 (32989 to 118387)	72.89 (37.5 to 134.59)	62.34 (-0.78 to 186.65)	0.02 (-0.3 to 0.34)
People's Republic of China	326179 (151540 to 529508)	48.91 (22.72 to 79.4)	181759 (105270 to 265299)	27.4 (15.87 to 40)	-44.28 (-65.75 to -3.75)	-2.53 (-2.95 to -2.12)

Country	Cases_1990	Rates_1990	Cases_2021	Rates_2021	Cases_change	EAPC_CI
Plurinational State of Bolivia	1772 (730 to 3372)	59.24 (24.41 to 112.77)	2873 (1321 to 4817)	45.91 (21.12 to 76.99)	62.17 (-15.76 to 246.63)	-1.06 (-1.41 to -0.71)
Portugal	3070 (2189 to 3949)	61.39 (43.77 to 78.96)	1394 (915 to 1883)	30.43 (19.98 to 41.11)	-54.6 (-64.7 to -43.6)	-2.03 (-2.24 to -1.81)
Principality of Andorra	13 (8 to 21)	40.5 (23.27 to 63.93)	10 (5 to 15)	23.05 (12.03 to 36.32)	-27.59 (-59.61 to 15.94)	-1.65 (-1.84 to -1.46)
Principality of Monaco	10 (6 to 14)	69.69 (43.46 to 104.79)	4 (2 to 7)	30.22 (16.83 to 50.86)	-55.8 (-74.08 to -23.8)	-2.67 (-2.79 to -2.54)
Puerto Rico	589 (353 to 853)	31.91 (19.14 to 46.22)	346 (216 to 474)	23.62 (14.76 to 32.38)	-41.21 (-59.66 to -13.81)	-1.51 (-1.74 to -1.29)
Republic of Albania	485 (304 to 777)	28.33 (17.75 to 45.41)	326 (210 to 459)	25.87 (16.65 to 36.45)	-32.81 (-57.37 to 5.73)	-0.32 (-0.68 to 0.04)
Republic of Angola	1094 (475 to 2267)	23.5 (10.21 to 48.71)	2829 (1207 to 5916)	19.3 (8.23 to 40.35)	158.72 (58.28 to 344.15)	-0.72 (-0.84 to -0.59)
Republic of Armenia	534 (368 to 687)	31.25 (21.56 to 40.24)	401 (286 to 518)	27.55 (19.65 to 35.6)	-24.81 (-37.92 to -9.52)	-1 (-1.59 to -0.4)
Republic of Austria	1967 (1366 to 2508)	48.86 (33.93 to 62.29)	873 (586 to 1184)	21.65 (14.52 to 29.36)	-55.59 (-66.84 to -44.33)	-2.42 (-2.64 to -2.19)
Republic of Azerbaijan	550 (306 to 817)	14.97 (8.34 to 22.27)	703 (434 to 1089)	12.63 (7.79 to 19.55)	28.01 (-21.46 to 104.91)	-1.25 (-1.67 to -0.82)
Republic of Belarus	2887 (1880 to 3817)	57.26 (37.29 to 75.7)	3135 (2248 to 4147)	73.69 (52.83 to 97.47)	8.59 (-23.56 to 56.68)	0.44 (0.18 to 0.71)
Republic of Benin	351 (134 to 869)	17.53 (6.67 to 43.39)	1171 (480 to 2759)	18.73 (7.68 to 44.14)	233.49 (126.78 to 441.63)	0.27 (0.01 to 0.54)
Republic of Botswana	99 (49 to 204)	16.36 (8.09 to 33.85)	188 (104 to 362)	13.79 (7.67 to 26.63)	90.19 (15.83 to 227.57)	-0.79 (-0.98 to -0.59)
Republic of Bulgaria	3159 (2234 to 4098)	76 (53.75 to 98.59)	2078 (1434 to 2729)	70.76 (48.82 to	-34.24 (-48.74 to -16)	-0.25 (-0.45 to -

Country	Cases_1990	Rates_1990	Cases_2021	Rates_2021	Cases_change	EAPC_CI
				92.92)		0.05)
Republic of Burundi	954 (292 to 2478)	39.32 (12.06 to 102.18)	1573 (410 to 5061)	25.19 (6.56 to 81.05)	64.92 (-10.22 to 215.52)	-2.04 (-2.36 to -1.73)
Republic of Cabo Verde	38 (14 to 102)	25.54 (9.54 to 69.01)	73 (32 to 180)	23.37 (10.16 to 57.38)	94.92 (30.33 to 206.31)	-0.34 (-0.56 to -0.13)
Republic of Cameroon	706 (259 to 1576)	15.6 (5.72 to 34.81)	4255 (1686 to 9224)	27.58 (10.93 to 59.79)	502.5 (262.95 to 948.79)	1.88 (1.19 to 2.58)
Republic of Chad	535 (153 to 1576)	21.47 (6.15 to 63.24)	1637 (542 to 4382)	22.07 (7.3 to 59.08)	206.11 (96.95 to 453.04)	0.05 (-0.18 to 0.28)
Republic of Chile	3642 (2436 to 4845)	51.22 (34.26 to 68.14)	3241 (1959 to 4448)	34.1 (20.61 to 46.81)	-11 (-33.97 to 11.4)	-1.28 (-1.58 to -0.99)
Republic of Colombia	7000 (4099 to 9968)	41.38 (24.23 to 58.93)	9848 (5277 to 14375)	37.71 (20.21 to 55.05)	40.7 (-5.22 to 110.89)	0.06 (-0.13 to 0.25)
Republic of Costa Rica	475 (288 to 668)	30.82 (18.68 to 43.35)	1042 (657 to 1428)	41.63 (26.26 to 57.05)	119.37 (62.36 to 206.88)	0.42 (0.2 to 0.65)
Republic of Côte d'Ivoire	1437 (591 to 3169)	25.83 (10.63 to 56.97)	3673 (1621 to 8457)	26.82 (11.84 to 61.76)	155.58 (54.51 to 334.41)	0.16 (0.01 to 0.31)
Republic of Croatia	1818 (1232 to 2420)	74.46 (50.45 to 99.15)	574 (391 to 747)	31.35 (21.37 to 40.8)	-68.44 (-76.32 to -57.76)	-2.73 (-3.14 to -2.33)
Republic of Cuba	2338 (1299 to 3517)	38 (21.11 to 57.16)	1246 (677 to 1854)	24.51 (13.31 to 36.47)	-46.72 (-67.48 to -12.52)	-1.47 (-1.71 to -1.23)
Republic of Cyprus	210 (135 to 300)	52.04 (33.4 to 74.2)	153 (93 to 214)	21.64 (13.12 to 30.28)	-27.29 (-50.45 to 4.83)	-3.5 (-3.74 to -3.26)
Republic of Djibouti	48 (18 to 123)	23.1 (8.53 to 59.14)	190 (72 to 452)	27.55 (10.36 to 65.49)	295.94 (138 to 576.34)	0.53 (0.31 to 0.76)
Republic of Ecuador	1386 (617 to 2393)	27.98 (12.45 to 48.29)	4486 (2610 to 6723)	47.76 (27.79 to 71.58)	223.6 (97.49 to 524.61)	2.71 (2.35 to 3.07)
Republic of El	1162 (633	46.43	1119 (633	33.81	-3.64 (-39.69	-0.84 (-

Country	Cases_1990	Rates_1990	Cases_2021	Rates_2021	Cases_change	EAPC_CI
Salvador	to 1825)	(25.31 to 72.95)	to 1701)	(19.13 to 51.38)	to 56.54)	1.41 to -0.27)
Republic of Equatorial Guinea	51 (22 to 112)	27.96 (11.92 to 61.78)	98 (49 to 167)	12.11 (6.07 to 20.6)	94.33 (0.35 to 296.03)	-3.38 (-3.98 to -2.77)
Republic of Estonia	517 (355 to 646)	68.14 (46.81 to 85.03)	157 (107 to 209)	27.38 (18.63 to 36.46)	-69.62 (-77.14 to -59.91)	-4.01 (-4.48 to -3.55)
Republic of Fiji	339 (213 to 512)	85.86 (53.79 to 129.69)	409 (255 to 590)	87.36 (54.43 to 126.03)	20.48 (-16.6 to 84.66)	0.07 (-0.02 to 0.17)
Republic of Finland	3513 (2615 to 4258)	136.06 (101.26 to 164.91)	958 (675 to 1210)	40.94 (28.85 to 51.74)	-72.74 (-77.61 to -68.09)	-4.17 (-4.39 to -3.94)
Republic of Ghana	2808 (1082 to 7375)	41.06 (15.82 to 107.86)	5725 (2469 to 12562)	32.67 (14.09 to 71.67)	103.92 (36.69 to 224.62)	-0.76 (-0.98 to -0.54)
Republic of Guatemala	420 (190 to 683)	11.88 (5.38 to 19.3)	2650 (1790 to 3524)	31.5 (21.28 to 41.88)	530.91 (321.09 to 993.11)	3.84 (3.32 to 4.36)
Republic of Guinea	587 (227 to 1457)	23.32 (9.02 to 57.86)	1592 (662 to 3560)	25.96 (10.79 to 58.07)	171.09 (72.14 to 360.89)	0.71 (0.55 to 0.86)
Republic of Guinea-Bissau	176 (59 to 398)	40.19 (13.51 to 91.1)	406 (141 to 949)	40.61 (14.06 to 94.91)	131.33 (36.39 to 293.78)	0.14 (0.1 to 0.18)
Republic of Haiti	3772 (1802 to 6152)	128.53 (61.42 to 209.64)	9230 (4154 to 15333)	134.64 (60.6 to 223.66)	144.71 (44.57 to 321.07)	0.42 (0.29 to 0.54)
Republic of Honduras	1503 (771 to 2432)	73.37 (37.63 to 118.72)	3407 (1628 to 5823)	62.94 (30.08 to 107.55)	126.75 (25.6 to 309.14)	-0.87 (-1.1 to -0.65)
Republic of Iceland	35 (21 to 50)	26.26 (15.85 to 37.44)	31 (19 to 44)	19.07 (11.53 to 26.51)	-9.65 (-34.12 to 26.21)	-1.2 (-1.36 to -1.04)
Republic of India	166682 (95781 to 266307)	39.65 (22.78 to 63.35)	239363 (145138 to 360428)	30.7 (18.62 to 46.23)	43.6 (15.21 to 83.8)	-0.86 (-1.04 to -0.69)
Republic of Indonesia	65254 (42713 to 95514)	69.04 (45.19 to 101.05)	103297 (68454 to 151099)	67.36 (44.64 to 98.54)	58.3 (24.21 to 112.73)	0.21 (0.01 to 0.41)

Country	Cases_1990	Rates_1990	Cases_2021	Rates_2021	Cases_change	EAPC_CI
Republic of Iraq	2816 (1709 to 4165)	33.26 (20.18 to 49.2)	5214 (3232 to 7571)	23.54 (14.59 to 34.17)	85.18 (13.84 to 195.24)	-1.55 (-1.77 to -1.32)
Republic of Italy	10033 (6963 to 12562)	34.93 (24.24 to 43.74)	3847 (2514 to 5175)	15.63 (10.22 to 21.03)	-61.65 (-69.85 to -53.51)	-2.48 (-2.75 to -2.21)
Republic of Kazakhstan	4912 (3171 to 6713)	59.78 (38.6 to 81.7)	5193 (3676 to 6786)	55.5 (39.3 to 72.53)	5.71 (-24.71 to 49.73)	-0.52 (-1 to -0.04)
Republic of Kenya	1220 (480 to 3012)	12.02 (4.73 to 29.66)	3972 (1753 to 9158)	15.19 (6.71 to 35.03)	225.54 (149.69 to 352.19)	1.03 (0.81 to 1.25)
Republic of Kiribati	34 (18 to 61)	93.33 (48.09 to 166.79)	75 (40 to 124)	120.35 (63.68 to 199.94)	118.26 (21.92 to 289.48)	0.56 (0.42 to 0.71)
Republic of Korea	21729 (12842 to 33157)	84.03 (49.66 to 128.22)	6637 (3600 to 10150)	27.33 (14.82 to 41.79)	-69.45 (-81.26 to -52.91)	-4.45 (-4.71 to -4.19)
Republic of Latvia	785 (543 to 1012)	61.05 (42.22 to 78.73)	335 (231 to 446)	42.55 (29.27 to 56.57)	-57.26 (-68.59 to -44.07)	-1.77 (-2.25 to -1.3)
Republic of Liberia	267 (97 to 680)	24.63 (8.94 to 62.75)	884 (279 to 2877)	31.52 (9.95 to 102.61)	231.3 (60.63 to 530.92)	0.44 (0.21 to 0.67)
Republic of Lithuania	1157 (821 to 1453)	63.18 (44.83 to 79.37)	560 (403 to 706)	48.48 (34.92 to 61.09)	-51.57 (-61.81 to -38.33)	-0.58 (-1.26 to 0.09)
Republic of Madagascar	2382 (823 to 6146)	44.91 (15.51 to 115.88)	6751 (2051 to 20572)	47.82 (14.53 to 145.7)	183.39 (75.25 to 360.01)	0.28 (0.19 to 0.37)
Republic of Malawi	1104 (378 to 2800)	25.07 (8.6 to 63.61)	2532 (777 to 7506)	26.15 (8.02 to 77.53)	129.44 (47.29 to 240.02)	-0.05 (-0.31 to 0.22)
Republic of Maldives	44 (25 to 66)	45.8 (26.31 to 69.8)	79 (48 to 120)	23.4 (14.08 to 35.48)	81.63 (17.53 to 191.54)	-2.43 (-2.81 to -2.05)
Republic of Mali	737 (244 to 1734)	20.38 (6.75 to 47.91)	1606 (657 to 3643)	15.29 (6.25 to 34.68)	117.79 (25.22 to 291.92)	-0.86 (-1.06 to -0.66)
Republic of Malta	57 (41 to 74)	29.97 (21.55 to 38.4)	38 (24 to 52)	19.71 (12.36 to 26.74)	-33.19 (-50.53 to -17.04)	-1.45 (-1.59 to -1.31)

Country	Cases_1990	Rates_1990	Cases_2021	Rates_2021	Cases_change	EAPC_CI
Republic of Mauritius	406 (269 to 541)	67.08 (44.45 to 89.34)	549 (372 to 718)	85.78 (58.2 to 112.23)	35.13 (8.54 to 72.49)	1.61 (1.16 to 2.06)
Republic of Moldova	667 (450 to 859)	30.35 (20.48 to 39.11)	538 (381 to 714)	30.03 (21.29 to 39.88)	-19.39 (-38.75 to 7.52)	-0.59 (-1.18 to 0.01)
Republic of Mozambique	2078 (719 to 5891)	35.95 (12.44 to 101.89)	6784 (2136 to 19439)	47.57 (14.97 to 136.3)	226.43 (110.9 to 414.64)	1.59 (1.31 to 1.88)
Republic of Namibia	86 (44 to 157)	13.08 (6.73 to 23.8)	193 (101 to 353)	14.93 (7.86 to 27.38)	123.75 (33.7 to 259.15)	0.09 (-0.24 to 0.43)
Republic of Nauru	9 (4 to 14)	173.97 (81.17 to 286.63)	11 (7 to 17)	194.19 (119.09 to 287.52)	29.07 (-21.54 to 124.56)	0.06 (-0.56 to 0.68)
Republic of Nicaragua	607 (366 to 880)	35.14 (21.16 to 50.91)	1108 (683 to 1587)	30.86 (19.02 to 44.19)	82.48 (18.96 to 175.92)	-0.19 (-0.36 to -0.01)
Republic of Niue	1 (1 to 2)	98.18 (53.26 to 169.2)	1 (0 to 1)	91.23 (56.33 to 142.71)	-29.38 (-60.18 to 21.75)	-0.74 (-0.95 to -0.53)
Republic of Palau	7 (3 to 11)	80.02 (38.39 to 130.56)	12 (7 to 17)	129.89 (74.56 to 189.95)	71.79 (1.92 to 227.18)	1.46 (1.22 to 1.69)
Republic of Panama	422 (251 to 599)	34.22 (20.37 to 48.64)	928 (546 to 1312)	42.89 (25.23 to 60.64)	120.17 (54.22 to 232.55)	1.22 (0.96 to 1.48)
Republic of Paraguay	1396 (892 to 1971)	73.83 (47.18 to 104.24)	1995 (1156 to 3069)	51.69 (29.96 to 79.53)	42.89 (-11.67 to 119.32)	-1.12 (-1.22 to -1.01)
Republic of Peru	2854 (1113 to 4806)	26.69 (10.41 to 44.96)	7592 (4203 to 11553)	39.28 (21.75 to 59.77)	166.05 (48.76 to 433.44)	1.86 (1.4 to 2.32)
Republic of Poland	17461 (12008 to 22182)	92.08 (63.32 to 116.97)	8582 (5991 to 10908)	47.88 (33.42 to 60.86)	-50.85 (-59.21 to -41.46)	-2.32 (-2.51 to -2.12)
Republic of Rwanda	1226 (412 to 2819)	38.85 (13.07 to 89.34)	1225 (396 to 3649)	17.87 (5.78 to 53.26)	-0.11 (-42.77 to 64.88)	-3.83 (-4.39 to -3.26)
Republic of San Marino	3 (2 to 4)	22.15 (14.18 to	2 (1 to 3)	12.06 (7.08 to 18.49)	-39.94 (-61.75 to -	-1.35 (-1.57 to -

Country	Cases_1990	Rates_1990	Cases_2021	Rates_2021	Cases_change	EAPC_CI
		31.04)			7.08)	1.14)
Republic of Senegal	921 (380 to 2065)	28.2 (11.65 to 63.24)	1788 (709 to 4504)	23.11 (9.16 to 58.22)	94.22 (30.27 to 194.71)	-0.45 (-0.69 to -0.21)
Republic of Serbia	5664 (3794 to 7695)	118.9 (79.63 to 161.51)	2501 (1683 to 3369)	58.99 (39.69 to 79.46)	-55.84 (-68.62 to -37.82)	-2.74 (-3.07 to -2.4)
Republic of Seychelles	18 (11 to 26)	47.72 (28.53 to 68.89)	18 (10 to 27)	33.08 (18.44 to 50.54)	1.31 (-26.98 to 32.05)	-0.91 (-1.04 to -0.78)
Republic of Sierra Leone	544 (220 to 1281)	28.65 (11.57 to 67.43)	1318 (523 to 3357)	29.63 (11.77 to 75.49)	142.06 (50.89 to 277.93)	0.19 (-0.02 to 0.4)
Republic of Singapore	560 (343 to 783)	29.46 (18.05 to 41.16)	344 (177 to 556)	11.54 (5.93 to 18.63)	-38.56 (-60 to -13.32)	-4.01 (-4.4 to -3.63)
Republic of Slovenia	461 (307 to 612)	45.45 (30.23 to 60.3)	157 (103 to 212)	17.91 (11.74 to 24.18)	-65.93 (-74.91 to -53.97)	-3.36 (-3.72 to -3)
Republic of South Africa	3681 (2432 to 5035)	19.51 (12.89 to 26.69)	5359 (3675 to 7291)	17.2 (11.8 to 23.4)	45.58 (11.43 to 87.96)	-0.46 (-0.82 to -0.1)
Republic of South Sudan	470 (206 to 1046)	17.42 (7.62 to 38.75)	895 (353 to 2078)	20.11 (7.94 to 46.71)	90.26 (5.04 to 248.97)	0.48 (0.1 to 0.86)
Republic of Sudan	4296 (1771 to 7316)	47.14 (19.44 to 80.28)	6214 (2889 to 11401)	27.79 (12.92 to 50.99)	44.65 (-14.87 to 140.02)	-1.85 (-1.91 to -1.79)
Republic of Suriname	76 (39 to 116)	38.54 (19.64 to 58.55)	117 (66 to 180)	40.81 (22.92 to 62.5)	53.82 (-10.1 to 161.71)	-0.04 (-0.22 to 0.15)
Republic of Tajikistan	731 (410 to 1142)	30.31 (17.01 to 47.39)	1471 (836 to 2469)	28.61 (16.27 to 48.03)	101.26 (14.45 to 265.09)	-0.86 (-1.13 to -0.59)
Republic of the Congo	280 (134 to 532)	25.3 (12.15 to 48.15)	696 (335 to 1405)	24.7 (11.88 to 49.85)	148.71 (46.3 to 342.37)	-0.31 (-0.48 to -0.14)
Republic of the Gambia	104 (41 to 245)	23.49 (9.32 to 55.29)	344 (128 to 909)	28.98 (10.75 to 76.67)	230.12 (94.53 to 393.47)	0.5 (0.25 to 0.75)
Republic of the Marshall	18 (8 to 33)	89.06 (41.67 to	37 (18 to	122.64 (59.29 to	109.19 (17.83	0.96 (0.88

Country	Cases_1990	Rates_1990	Cases_2021	Rates_2021	Cases_change	EAPC_CI
Islands		162.16)	64)	210.91)	to 259.76)	to 1.04)
Republic of the Niger	697 (237 to 1723)	20.82 (7.07 to 51.44)	1677 (613 to 4487)	16.17 (5.91 to 43.26)	140.58 (51.18 to 301.66)	-0.58 (-0.73 to -0.42)
Republic of the Philippines	9325 (5669 to 13203)	29.98 (18.22 to 42.44)	24261 (14878 to 34505)	40.41 (24.78 to 57.48)	160.17 (90.04 to 250.64)	1.46 (1.28 to 1.64)
Republic of the Union of Myanmar	14009 (7101 to 23605)	68.61 (34.78 to 115.62)	16867 (10111 to 26138)	57.37 (34.39 to 88.91)	20.4 (-25.06 to 107.22)	-0.4 (-0.7 to -0.1)
Republic of Trinidad and Tobago	252 (144 to 363)	40.75 (23.24 to 58.63)	557 (352 to 810)	80.75 (51.01 to 117.44)	121.05 (47.58 to 239.43)	2 (1.78 to 2.22)
Republic of Tunisia	742 (374 to 1310)	18.12 (9.14 to 31.97)	1033 (533 to 1694)	17.09 (8.82 to 28.03)	39.15 (-22.32 to 142.5)	-0.44 (-0.52 to -0.36)
Republic of Turkey	18699 (10429 to 29462)	64.52 (35.98 to 101.65)	12741 (7188 to 18411)	29 (16.36 to 41.9)	-31.86 (-57.93 to 13.07)	-2.9 (-3.02 to -2.79)
Republic of Uganda	1566 (479 to 3955)	21.08 (6.45 to 53.22)	3115 (1086 to 7781)	15.5 (5.4 to 38.71)	98.9 (29.48 to 224.27)	-1.82 (-2.12 to -1.52)
Republic of Uzbekistan	1456 (840 to 2087)	14.88 (8.59 to 21.34)	5350 (3695 to 7014)	30 (20.72 to 39.33)	267.52 (168.27 to 433.94)	2.51 (2.06 to 2.96)
Republic of Vanuatu	97 (54 to 160)	138.66 (76.89 to 227.38)	222 (126 to 372)	142.39 (80.55 to 238.57)	128.17 (34.85 to 262.83)	-0.2 (-0.29 to -0.11)
Republic of Yemen	1719 (729 to 3221)	31.59 (13.4 to 59.2)	4225 (1851 to 8250)	25.26 (11.07 to 49.33)	145.83 (36.61 to 356.69)	-1.09 (-1.35 to -0.83)
Republic of Zambia	533 (165 to 1436)	15.08 (4.67 to 40.63)	2345 (875 to 5526)	24.27 (9.05 to 57.2)	340.17 (141.18 to 754.56)	1.79 (1.66 to 1.91)
Republic of Zimbabwe	658 (379 to 1106)	14.27 (8.22 to 23.99)	3190 (1656 to 5517)	41.18 (21.38 to 71.23)	385 (207 to 648.2)	4.36 (3.53 to 5.19)
Romania	7043 (4855 to 9070)	62.12 (42.82 to 80)	5169 (3569 to 6768)	62.05 (42.85 to 81.24)	-26.61 (-43.14 to -5.8)	-0.36 (-0.87 to 0.15)
Russian	52466	70.69	59076	87.6 (62.6	12.6 (2.47 to	-0.34 (-

Country	Cases_1990	Rates_1990	Cases_2021	Rates_2021	Cases_change	EAPC_CI
Federation	(37933 to 65219)	(51.11 to 87.87)	(42216 to 73314)	to 108.71)	22.03)	0.99 to 0.31)
Saint Kitts and Nevis	14 (8 to 20)	69.88 (41.63 to 102.66)	15 (10 to 21)	47.32 (30.29 to 68.03)	7.52 (-29.08 to 74.26)	-2.06 (-2.45 to -1.67)
Saint Lucia	33 (21 to 45)	49.39 (31.5 to 67.65)	40 (24 to 57)	43.61 (26.21 to 61.29)	22.63 (-6.9 to 63.67)	-0.31 (-0.55 to -0.06)
Saint Vincent and the Grenadines	15 (9 to 22)	28.03 (16.33 to 41.34)	23 (14 to 32)	40.11 (24.23 to 55.78)	51.31 (4.7 to 133.92)	1 (0.67 to 1.33)
Slovak Republic	1581 (1059 to 2166)	59.19 (39.63 to 81.08)	895 (599 to 1239)	34.6 (23.16 to 47.92)	-43.4 (-59.37 to -17.84)	-1.58 (-1.71 to -1.45)
Socialist Republic of Viet Nam	12444 (6439 to 24919)	37.95 (19.64 to 76.01)	24984 (14423 to 40813)	47.97 (27.69 to 78.37)	100.78 (15.59 to 264.83)	1.31 (1.07 to 1.54)
Solomon Islands	78 (31 to 157)	51.26 (20.4 to 103.93)	241 (114 to 435)	69.89 (33.14 to 126.42)	209.9 (58.93 to 560.47)	1.24 (1.08 to 1.4)
State of Eritrea	507 (139 to 1317)	32.92 (9 to 85.43)	977 (289 to 2475)	28.55 (8.45 to 72.31)	92.63 (12.58 to 241.74)	-0.31 (-0.54 to -0.09)
State of Israel	561 (383 to 738)	23.09 (15.78 to 30.39)	476 (306 to 643)	10.58 (6.82 to 14.32)	-15.17 (-34.14 to 6.14)	-2.54 (-2.71 to -2.36)
State of Kuwait	139 (81 to 200)	13.53 (7.91 to 19.5)	250 (158 to 359)	8.2 (5.19 to 11.79)	80.05 (32.55 to 148.67)	-2.07 (-2.54 to -1.59)
State of Libya	350 (201 to 549)	17.71 (10.15 to 27.76)	1126 (554 to 1848)	27.31 (13.44 to 44.84)	221.29 (85.49 to 403.94)	1.77 (1.56 to 1.99)
State of Qatar	105 (60 to 180)	36.12 (20.56 to 61.78)	392 (214 to 591)	17.86 (9.76 to 26.96)	272.11 (101.11 to 541.62)	-3.13 (-3.58 to -2.67)
Sultanate of Oman	163 (83 to 290)	16.33 (8.32 to 29.19)	430 (269 to 650)	14.37 (8.98 to 21.7)	164.81 (59.38 to 356.02)	-0.14 (-0.36 to 0.08)
Swiss Confederation	1290 (846 to 1725)	35.55 (23.33 to 47.56)	388 (225 to 552)	9.68 (5.62 to 13.79)	-69.93 (-78.41 to -60.55)	-4.51 (-4.79 to -4.23)

Country	Cases_1990	Rates_1990	Cases_2021	Rates_2021	Cases_change	EAPC_CI
Syrian Arab Republic	1479 (751 to 2402)	26.39 (13.41 to 42.87)	1440 (848 to 2226)	20.28 (11.94 to 31.36)	-2.64 (-49.86 to 76.63)	-0.73 (-1.21 to -0.25)
Taiwan (Province of China)	2327 (1444 to 3278)	20.67 (12.83 to 29.11)	2786 (1557 to 4006)	24.5 (13.69 to 35.23)	19.71 (-18 to 65.31)	-0.12 (-0.41 to 0.18)
Togolese Republic	445 (168 to 1062)	27.65 (10.45 to 66.04)	1177 (431 to 3012)	28.32 (10.38 to 72.47)	164.66 (61.92 to 306.83)	0.06 (-0.2 to 0.31)
Tokelau	1 (0 to 1)	79.39 (34.54 to 150.46)	1 (0 to 1)	94.75 (54.2 to 158.04)	12.2 (-34.87 to 111.55)	0.03 (-0.17 to 0.24)
Turkmenistan	800 (493 to 1110)	45.53 (28.04 to 63.12)	2488 (1535 to 3729)	92.96 (57.35 to 139.32)	210.85 (116.41 to 355.58)	2.47 (2.04 to 2.91)
Tuvalu	5 (2 to 10)	115.35 (47.44 to 216.09)	8 (4 to 13)	125.04 (67.23 to 205.92)	49.98 (-11.27 to 174.67)	0.03 (-0.19 to 0.24)
Ukraine	13581 (9458 to 17721)	54.43 (37.91 to 71.02)	16152 (10149 to 23050)	79.86 (50.18 to 113.96)	18.93 (-18.45 to 78.17)	1.1 (0.94 to 1.26)
Union of the Comoros	54 (22 to 116)	26.59 (10.77 to 56.84)	95 (39 to 238)	24.32 (9.96 to 60.65)	74.88 (5.65 to 208.83)	-0.6 (-0.99 to -0.21)
United Arab Emirates	379 (216 to 584)	32.16 (18.35 to 49.51)	1333 (810 to 2047)	19.42 (11.8 to 29.82)	251.6 (121.93 to 524.07)	-1.94 (-2.39 to -1.48)
United Kingdom of Great Britain and Northern Ireland	21474 (15944 to 26402)	75.45 (56.02 to 92.77)	7791 (5512 to 9960)	25.68 (18.17 to 32.82)	-63.72 (-67.03 to -60.74)	-4.01 (-4.28 to -3.74)
United Mexican States	10398 (6445 to 14857)	24.45 (15.15 to 34.93)	25009 (16243 to 33769)	36.53 (23.72 to 49.32)	140.51 (71.08 to 244.81)	1.36 (1.27 to 1.45)
United Republic of Tanzania	2232 (764 to 6089)	19.74 (6.76 to 53.85)	5325 (2042 to 15182)	18.88 (7.24 to 53.84)	138.53 (59.74 to 263.87)	-0.33 (-0.5 to -0.16)
United States of America	48929 (31483 to 66590)	36.45 (23.45 to 49.6)	38994 (23670 to 53769)	25.65 (15.57 to 35.37)	-20.3 (-34.35 to -3.34)	-1.52 (-1.83 to -1.19)

Country	Cases_1990	Rates_1990	Cases_2021	Rates_2021	Cases_change	EAPC_CI
United States Virgin Islands	32 (17 to 53)	58.13 (31.81 to 95.83)	14 (7 to 22)	40.86 (21.21 to 63.72)	-56.7 (-76.42 to -27.66)	-0.63 (-0.8 to -0.46)

Supplementary Table S4. Projected subarachnoid hemorrhage deaths and DALYs attributable to high systolic blood pressure among 25–49 years from 1990 to 2050, by sex, age and SDI region.

Year	Region	Measure	Type	Actual	Forecast	Lower_95	Upper_95
**SDI regions groups**							
1990	Global	Deaths	Actual	0.90			
1995	Global	Deaths	Actual	0.95			
2000	Global	Deaths	Actual	0.90			
2005	Global	Deaths	Actual	0.77			
2010	Global	Deaths	Actual	0.72			
2015	Global	Deaths	Actual	0.68			
2020	Global	Deaths	Actual	0.64			
2025	Global	Deaths	Forecast		0.63	0.56	0.69
2030	Global	Deaths	Forecast		0.63	0.47	0.78
2035	Global	Deaths	Forecast		0.63	0.41	0.85
2040	Global	Deaths	Forecast		0.63	0.35	0.90
2045	Global	Deaths	Forecast		0.63	0.30	0.95
2050	Global	Deaths	Forecast		0.63	0.26	0.99
1990	High SDI	Deaths	Actual	1.06			
1995	High SDI	Deaths	Actual	1.02			
2000	High SDI	Deaths	Actual	0.91			
2005	High SDI	Deaths	Actual	0.75			
2010	High SDI	Deaths	Actual	0.59			
2015	High SDI	Deaths	Actual	0.53			
2020	High SDI	Deaths	Actual	0.49			
2025	High SDI	Deaths	Forecast		0.43	0.36	0.50
2030	High SDI	Deaths	Forecast		0.34	0.20	0.49
2035	High SDI	Deaths	Forecast		0.26	0.06	0.45
2040	High SDI	Deaths	Forecast		0.17	-0.07	0.41
2045	High SDI	Deaths	Forecast		0.09	-0.19	0.36
2050	High SDI	Deaths	Foreca		0.00	-0.30	0.31

			st				
1990	High-middle SDI	Deaths	Actual	0.91			
1995	High-middle SDI	Deaths	Actual	1.10			
2000	High-middle SDI	Deaths	Actual	1.08			
2005	High-middle SDI	Deaths	Actual	0.88			
2010	High-middle SDI	Deaths	Actual	0.77			
2015	High-middle SDI	Deaths	Actual	0.69			
2020	High-middle SDI	Deaths	Actual	0.62			
2025	High-middle SDI	Deaths	Forecast		0.60	0.38	0.81
2030	High-middle SDI	Deaths	Forecast		0.58	-0.08	1.24
2035	High-middle SDI	Deaths	Forecast		0.55	-0.69	1.80
2040	High-middle SDI	Deaths	Forecast		0.53	-1.42	2.48
2045	High-middle SDI	Deaths	Forecast		0.51	-2.23	3.25
2050	High-middle SDI	Deaths	Forecast		0.49	-3.14	4.11
1990	Middle SDI	Deaths	Actual	0.95			
1995	Middle SDI	Deaths	Actual	0.99			
2000	Middle SDI	Deaths	Actual	0.93			
2005	Middle SDI	Deaths	Actual	0.77			
2010	Middle SDI	Deaths	Actual	0.76			
2015	Middle SDI	Deaths	Actual	0.73			
2020	Middle SDI	Deaths	Actual	0.69			
2025	Middle SDI	Deaths	Forecast		0.68	0.61	0.74
2030	Middle SDI	Deaths	Forecast		0.67	0.51	0.83
2035	Middle SDI	Deaths	Forecast		0.67	0.42	0.92
2040	Middle SDI	Deaths	Forecast		0.67	0.35	1.00
2045	Middle SDI	Deaths	Forecast		0.67	0.28	1.06

2050	Middle SDI	Deaths	Forecast		0.67	0.22	1.12
1990	Low-middle SDI	Deaths	Actual	0.83			
1995	Low-middle SDI	Deaths	Actual	0.83			
2000	Low-middle SDI	Deaths	Actual	0.83			
2005	Low-middle SDI	Deaths	Actual	0.82			
2010	Low-middle SDI	Deaths	Actual	0.81			
2015	Low-middle SDI	Deaths	Actual	0.77			
2020	Low-middle SDI	Deaths	Actual	0.74			
2025	Low-middle SDI	Deaths	Forecast		0.71	0.68	0.74
2030	Low-middle SDI	Deaths	Forecast		0.67	0.61	0.74
2035	Low-middle SDI	Deaths	Forecast		0.64	0.54	0.75
2040	Low-middle SDI	Deaths	Forecast		0.61	0.46	0.76
2045	Low-middle SDI	Deaths	Forecast		0.58	0.38	0.77
2050	Low-middle SDI	Deaths	Forecast		0.54	0.29	0.79
1990	Low SDI	Deaths	Actual	0.54			
1995	Low SDI	Deaths	Actual	0.54			
2000	Low SDI	Deaths	Actual	0.53			
2005	Low SDI	Deaths	Actual	0.51			
2010	Low SDI	Deaths	Actual	0.49			
2015	Low SDI	Deaths	Actual	0.47			
2020	Low SDI	Deaths	Actual	0.46			
2025	Low SDI	Deaths	Forecast		0.45	0.43	0.47
2030	Low SDI	Deaths	Forecast		0.44	0.40	0.47
2035	Low SDI	Deaths	Forecast		0.42	0.38	0.47
2040	Low SDI	Deaths	Forecast		0.41	0.36	0.46
2045	Low SDI	Deaths	Foreca		0.40	0.34	0.46

			st				
2050	Low SDI	Deaths	Forecast		0.39	0.32	0.45
1990	Global	DALYs	Actual	48.03			
1995	Global	DALYs	Actual	50.11			
2000	Global	DALYs	Actual	47.77			
2005	Global	DALYs	Actual	41.75			
2010	Global	DALYs	Actual	39.11			
2015	Global	DALYs	Actual	36.92			
2020	Global	DALYs	Actual	34.99			
2025	Global	DALYs	Forecast		34.78	31.53	38.03
2030	Global	DALYs	Forecast		34.78	27.32	42.23
2035	Global	DALYs	Forecast		34.78	24.03	45.53
2040	Global	DALYs	Forecast		34.78	21.38	48.18
2045	Global	DALYs	Forecast		34.78	19.14	50.42
2050	Global	DALYs	Forecast		34.78	17.17	52.38
1990	High SDI	DALYs	Actual	55.80			
1995	High SDI	DALYs	Actual	53.61			
2000	High SDI	DALYs	Actual	47.72			
2005	High SDI	DALYs	Actual	39.73			
2010	High SDI	DALYs	Actual	32.10			
2015	High SDI	DALYs	Actual	29.48			
2020	High SDI	DALYs	Actual	27.67			
2025	High SDI	DALYs	Forecast		24.61	21.09	28.14
2030	High SDI	DALYs	Forecast		20.64	13.37	27.91
2035	High SDI	DALYs	Forecast		16.48	6.36	26.59
2040	High SDI	DALYs	Forecast		12.28	-0.12	24.68
2045	High SDI	DALYs	Forecast		8.07	-6.27	22.41
2050	High SDI	DALYs	Forecast		3.86	-12.19	19.91
1990	High-middle SDI	DALYs	Actual	49.12			

1995	High-middle SDI	DALYs	Actual	58.39			
2000	High-middle SDI	DALYs	Actual	57.37			
2005	High-middle SDI	DALYs	Actual	47.58			
2010	High-middle SDI	DALYs	Actual	42.50			
2015	High-middle SDI	DALYs	Actual	38.41			
2020	High-middle SDI	DALYs	Actual	35.26			
2025	High-middle SDI	DALYs	Forecast		34.41	23.94	44.88
2030	High-middle SDI	DALYs	Forecast		33.57	1.30	65.83
2035	High-middle SDI	DALYs	Forecast		32.72	-28.16	93.60
2040	High-middle SDI	DALYs	Forecast		31.87	-63.10	126.85
2045	High-middle SDI	DALYs	Forecast		31.03	-102.74	164.80
2050	High-middle SDI	DALYs	Forecast		30.18	-146.58	206.94
1990	Middle SDI	DALYs	Actual	50.18			
1995	Middle SDI	DALYs	Actual	52.45			
2000	Middle SDI	DALYs	Actual	49.41			
2005	Middle SDI	DALYs	Actual	41.93			
2010	Middle SDI	DALYs	Actual	41.11			
2015	Middle SDI	DALYs	Actual	39.79			
2020	Middle SDI	DALYs	Actual	37.76			
2025	Middle SDI	DALYs	Forecast		37.46	34.34	40.59
2030	Middle SDI	DALYs	Forecast		37.32	29.62	45.02
2035	Middle SDI	DALYs	Forecast		37.25	25.28	49.22
2040	Middle SDI	DALYs	Forecast		37.21	21.47	52.95
2045	Middle SDI	DALYs	Forecast		37.19	18.13	56.25
2050	Middle SDI	DALYs	Forecast		37.19	15.18	59.19
1990	Low-middle	DALYs	Actual	44.45			

	SDI						
1995	Low-middle SDI	DALYs	Actual	44.15			
2000	Low-middle SDI	DALYs	Actual	44.39			
2005	Low-middle SDI	DALYs	Actual	43.79			
2010	Low-middle SDI	DALYs	Actual	43.22			
2015	Low-middle SDI	DALYs	Actual	41.37			
2020	Low-middle SDI	DALYs	Actual	40.04			
2025	Low-middle SDI	DALYs	Forecast		38.58	37.05	40.10
2030	Low-middle SDI	DALYs	Forecast		37.10	33.98	40.21
2035	Low-middle SDI	DALYs	Forecast		35.62	30.69	40.56
2040	Low-middle SDI	DALYs	Forecast		34.15	27.16	41.13
2045	Low-middle SDI	DALYs	Forecast		32.67	23.42	41.92
2050	Low-middle SDI	DALYs	Forecast		31.20	19.49	42.90
1990	Low SDI	DALYs	Actual	28.91			
1995	Low SDI	DALYs	Actual	29.01			
2000	Low SDI	DALYs	Actual	28.85			
2005	Low SDI	DALYs	Actual	27.80			
2010	Low SDI	DALYs	Actual	27.05			
2015	Low SDI	DALYs	Actual	26.01			
2020	Low SDI	DALYs	Actual	25.40			
2025	Low SDI	DALYs	Forecast		24.84	23.76	25.92
2030	Low SDI	DALYs	Forecast		24.27	22.58	25.95
2035	Low SDI	DALYs	Forecast		23.69	21.56	25.82
2040	Low SDI	DALYs	Forecast		23.12	20.62	25.61
2045	Low SDI	DALYs	Forecast		22.55	19.73	25.36
2050	Low SDI	DALYs	Forecast		21.97	18.87	25.07

Sex groups

1990	Male	DALYs	Actual	56.30			
1995	Male	DALYs	Actual	59.13			
2000	Male	DALYs	Actual	57.11			
2005	Male	DALYs	Actual	50.74			
2010	Male	DALYs	Actual	48.32			
2015	Male	DALYs	Actual	45.55			
2020	Male	DALYs	Actual	42.73			
2025	Male	DALYs	Forecast		42.22	38.42	46.03
2030	Male	DALYs	Forecast		42.17	33.88	50.46
2035	Male	DALYs	Forecast		42.16	30.49	53.83
2040	Male	DALYs	Forecast		42.16	27.78	56.54
2045	Male	DALYs	Forecast		42.16	25.49	58.82
2050	Male	DALYs	Forecast		42.16	23.48	60.84
1990	Female	DALYs	Actual	39.54			
1995	Female	DALYs	Actual	40.87			
2000	Female	DALYs	Actual	38.23			
2005	Female	DALYs	Actual	32.58			
2010	Female	DALYs	Actual	29.73			
2015	Female	DALYs	Actual	28.11			
2020	Female	DALYs	Actual	27.05			
2025	Female	DALYs	Forecast		26.72	24.35	29.08
2030	Female	DALYs	Forecast		26.52	20.45	32.60
2035	Female	DALYs	Forecast		26.42	16.69	36.14
2040	Female	DALYs	Forecast		26.35	13.28	39.43
2045	Female	DALYs	Forecast		26.32	10.22	42.42
2050	Female	DALYs	Forecast		26.30	7.46	45.13
1990	Both	DALYs	Actual	48.03			
1995	Both	DALYs	Actual	50.11			
2000	Both	DALYs	Actual	47.77			
2005	Both	DALYs	Actual	41.75			

2010	Both	DALYs	Actual	39.11			
2015	Both	DALYs	Actual	36.92			
2020	Both	DALYs	Actual	34.99			
2025	Both	DALYs	Forecast		34.78	31.53	38.03
2030	Both	DALYs	Forecast		34.78	27.32	42.23
2035	Both	DALYs	Forecast		34.78	24.03	45.53
2040	Both	DALYs	Forecast		34.78	21.38	48.18
2045	Both	DALYs	Forecast		34.78	19.14	50.42
2050	Both	DALYs	Forecast		34.78	17.17	52.38
1990	Male	Deaths	Actual	1.06			
1995	Male	Deaths	Actual	1.12			
2000	Male	Deaths	Actual	1.08			
2005	Male	Deaths	Actual	0.95			
2010	Male	Deaths	Actual	0.90			
2015	Male	Deaths	Actual	0.84			
2020	Male	Deaths	Actual	0.79			
2025	Male	Deaths	Forecast		0.77	0.69	0.85
2030	Male	Deaths	Forecast		0.77	0.60	0.94
2035	Male	Deaths	Forecast		0.77	0.53	1.01
2040	Male	Deaths	Forecast		0.77	0.47	1.06
2045	Male	Deaths	Forecast		0.77	0.42	1.11
2050	Male	Deaths	Forecast		0.77	0.38	1.15
1990	Female	Deaths	Actual	0.74			
1995	Female	Deaths	Actual	0.77			
2000	Female	Deaths	Actual	0.71			
2005	Female	Deaths	Actual	0.60			
2010	Female	Deaths	Actual	0.54			
2015	Female	Deaths	Actual	0.51			
2020	Female	Deaths	Actual	0.48			
2025	Female	Deaths	Forecast		0.46	0.41	0.51

2030	Female	Deaths	Forecast		0.42	0.32	0.53
2035	Female	Deaths	Forecast		0.39	0.25	0.52
2040	Female	Deaths	Forecast		0.35	0.18	0.51
2045	Female	Deaths	Forecast		0.31	0.12	0.50
2050	Female	Deaths	Forecast		0.27	0.06	0.48
1990	Both	Deaths	Actual	0.90			
1995	Both	Deaths	Actual	0.95			
2000	Both	Deaths	Actual	0.90			
2005	Both	Deaths	Actual	0.77			
2010	Both	Deaths	Actual	0.72			
2015	Both	Deaths	Actual	0.68			
2020	Both	Deaths	Actual	0.64			
2025	Both	Deaths	Forecast		0.63	0.56	0.69
2030	Both	Deaths	Forecast		0.63	0.47	0.78
2035	Both	Deaths	Forecast		0.63	0.41	0.85
2040	Both	Deaths	Forecast		0.63	0.35	0.90
2045	Both	Deaths	Forecast		0.63	0.30	0.95
2050	Both	Deaths	Forecast		0.63	0.26	0.99
1990	25-29 years	DALYs	Actual	24.66			
1995	25-29 years	DALYs	Actual	23.93			
2000	25-29 years	DALYs	Actual	22.74			
2005	25-29 years	DALYs	Actual	20.98			
2010	25-29 years	DALYs	Actual	20.01			
2015	25-29 years	DALYs	Actual	17.94			
2020	25-29 years	DALYs	Actual	17.10			
2025	25-29 years	DALYs	Forecast		16.09	15.40	16.78
2030	25-29 years	DALYs	Forecast		14.85	13.92	15.78
2035	25-29 years	DALYs	Forecast		13.62	12.50	14.74
2040	25-29 years	DALYs	Foreca		12.38	11.10	13.66

			st				
2045	25-29 years	DALYs	Forecast		11.15	9.73	12.57
2050	25-29 years	DALYs	Forecast		9.91	8.36	11.46
1990	30-34 years	DALYs	Actual	41.19			
1995	30-34 years	DALYs	Actual	39.76			
2000	30-34 years	DALYs	Actual	37.32			
2005	30-34 years	DALYs	Actual	33.90			
2010	30-34 years	DALYs	Actual	32.59			
2015	30-34 years	DALYs	Actual	29.79			
2020	30-34 years	DALYs	Actual	27.62			
2025	30-34 years	DALYs	Forecast		26.33	24.92	27.75
2030	30-34 years	DALYs	Forecast		24.23	21.99	26.46
2035	30-34 years	DALYs	Forecast		22.12	19.29	24.95
2040	30-34 years	DALYs	Forecast		20.01	16.70	23.33
2045	30-34 years	DALYs	Forecast		17.91	14.17	21.65
2050	30-34 years	DALYs	Forecast		15.80	11.68	19.92
1990	35-39 years	DALYs	Actual	71.25			
1995	35-39 years	DALYs	Actual	70.23			
2000	35-39 years	DALYs	Actual	64.34			
2005	35-39 years	DALYs	Actual	55.28			
2010	35-39 years	DALYs	Actual	51.45			
2015	35-39 years	DALYs	Actual	48.57			
2020	35-39 years	DALYs	Actual	44.74			
2025	35-39 years	DALYs	Forecast		41.99	37.90	46.07
2030	35-39 years	DALYs	Forecast		37.92	31.09	44.75
2035	35-39 years	DALYs	Forecast		33.85	25.10	42.61
2040	35-39 years	DALYs	Forecast		29.79	19.47	40.11
2045	35-39 years	DALYs	Forecast		25.72	14.04	37.41
2050	35-39 years	DALYs	Forecast		21.66	8.76	34.56

1990	40-44 years	DALYs	Actual	128.32			
1995	40-44 years	DALYs	Actual	127.09			
2000	40-44 years	DALYs	Actual	115.28			
2005	40-44 years	DALYs	Actual	98.05			
2010	40-44 years	DALYs	Actual	87.03			
2015	40-44 years	DALYs	Actual	81.23			
2020	40-44 years	DALYs	Actual	77.22			
2025	40-44 years	DALYs	Forecast		72.27	65.72	78.82
2030	40-44 years	DALYs	Forecast		65.43	51.56	79.30
2035	40-44 years	DALYs	Forecast		58.14	38.59	77.70
2040	40-44 years	DALYs	Forecast		50.75	26.61	74.89
2045	40-44 years	DALYs	Forecast		43.33	15.30	71.36
2050	40-44 years	DALYs	Forecast		35.90	4.45	67.35
1990	45-49 years	DALYs	Actual	178.85			
1995	45-49 years	DALYs	Actual	176.02			
2000	45-49 years	DALYs	Actual	160.34			
2005	45-49 years	DALYs	Actual	136.04			
2010	45-49 years	DALYs	Actual	120.47			
2015	45-49 years	DALYs	Actual	108.73			
2020	45-49 years	DALYs	Actual	101.07			
2025	45-49 years	DALYs	Forecast		93.67	85.08	102.27
2030	45-49 years	DALYs	Forecast		83.08	64.89	101.27
2035	45-49 years	DALYs	Forecast		71.72	46.08	97.36
2040	45-49 years	DALYs	Forecast		60.18	28.53	91.83

2045	45-49 years	DALYs	Forecast		48.60	11.86	85.35
2050	45-49 years	DALYs	Forecast		37.01	-4.22	78.25
1990	25-49 years	DALYs	Actual	48.03			
1995	25-49 years	DALYs	Actual	50.11			
2000	25-49 years	DALYs	Actual	47.77			
2005	25-49 years	DALYs	Actual	41.75			
2010	25-49 years	DALYs	Actual	39.11			
2015	25-49 years	DALYs	Actual	36.92			
2020	25-49 years	DALYs	Actual	34.99			
2025	25-49 years	DALYs	Forecast		34.78	31.53	38.03
2030	25-49 years	DALYs	Forecast		34.78	27.32	42.23
2035	25-49 years	DALYs	Forecast		34.78	24.03	45.53
2040	25-49 years	DALYs	Forecast		34.78	21.38	48.18
2045	25-49 years	DALYs	Forecast		34.78	19.14	50.42
2050	25-49 years	DALYs	Forecast		34.78	17.17	52.38
1990	25-29 years	Deaths	Actual	0.35			
1995	25-29 years	Deaths	Actual	0.34			
2000	25-29 years	Deaths	Actual	0.32			
2005	25-29 years	Deaths	Actual	0.29			
2010	25-29 years	Deaths	Actual	0.28			
2015	25-29 years	Deaths	Actual	0.25			
2020	25-29 years	Deaths	Actual	0.23			
2025	25-29 years	Deaths	Forecast		0.22	0.21	0.23
2030	25-29 years	Deaths	Forecast		0.20	0.18	0.21
2035	25-29 years	Deaths	Forecast		0.18	0.16	0.20
2040	25-29 years	Deaths	Forecast		0.16	0.14	0.18
2045	25-29 years	Deaths	Forecast		0.14	0.12	0.16
2050	25-29 years	Deaths	Forecast		0.12	0.10	0.14
1990	30-34 years	Deaths	Actual	0.64			

1995	30-34 years	Deaths	Actual	0.62			
2000	30-34 years	Deaths	Actual	0.58			
2005	30-34 years	Deaths	Actual	0.52			
2010	30-34 years	Deaths	Actual	0.50			
2015	30-34 years	Deaths	Actual	0.45			
2020	30-34 years	Deaths	Actual	0.41			
2025	30-34 years	Deaths	Forecast		0.39	0.37	0.41
2030	30-34 years	Deaths	Forecast		0.35	0.32	0.39
2035	30-34 years	Deaths	Forecast		0.32	0.27	0.37
2040	30-34 years	Deaths	Forecast		0.28	0.23	0.34
2045	30-34 years	Deaths	Forecast		0.25	0.18	0.31
2050	30-34 years	Deaths	Forecast		0.21	0.14	0.28
1990	35-39 years	Deaths	Actual	1.24			
1995	35-39 years	Deaths	Actual	1.22			
2000	35-39 years	Deaths	Actual	1.11			
2005	35-39 years	Deaths	Actual	0.94			
2010	35-39 years	Deaths	Actual	0.87			
2015	35-39 years	Deaths	Actual	0.81			
2020	35-39 years	Deaths	Actual	0.74			
2025	35-39 years	Deaths	Forecast		0.69	0.61	0.76
2030	35-39 years	Deaths	Forecast		0.61	0.48	0.74
2035	35-39 years	Deaths	Forecast		0.54	0.37	0.70
2040	35-39 years	Deaths	Forecast		0.46	0.27	0.65
2045	35-39 years	Deaths	Forecast		0.38	0.17	0.60
2050	35-39 years	Deaths	Forecast		0.31	0.07	0.55
1990	40-44 years	Deaths	Actual	2.49			
1995	40-44 years	Deaths	Actual	2.47			
2000	40-44 years	Deaths	Actual	2.23			
2005	40-44 years	Deaths	Actual	1.87			
2010	40-44 years	Deaths	Actual	1.65			
2015	40-44 years	Deaths	Actual	1.53			

2020	40-44 years	Deaths	Actual	1.44			
2025	40-44 years	Deaths	Forecast		1.34	1.20	1.48
2030	40-44 years	Deaths	Forecast		1.20	0.91	1.49
2035	40-44 years	Deaths	Forecast		1.05	0.65	1.45
2040	40-44 years	Deaths	Forecast		0.90	0.40	1.39
2045	40-44 years	Deaths	Forecast		0.74	0.17	1.32
2050	40-44 years	Deaths	Forecast		0.59	-0.05	1.24
1990	45-49 years	Deaths	Actual	3.86			
1995	45-49 years	Deaths	Actual	3.78			
2000	45-49 years	Deaths	Actual	3.43			
2005	45-49 years	Deaths	Actual	2.88			
2010	45-49 years	Deaths	Actual	2.53			
2015	45-49 years	Deaths	Actual	2.26			
2020	45-49 years	Deaths	Actual	2.09			
2025	45-49 years	Deaths	Forecast		1.90	1.69	2.11
2030	45-49 years	Deaths	Forecast		1.63	1.25	2.01
2035	45-49 years	Deaths	Forecast		1.36	0.86	1.86
2040	45-49 years	Deaths	Forecast		1.08	0.49	1.68
2045	45-49 years	Deaths	Forecast		0.81	0.14	1.49
2050	45-49 years	Deaths	Forecast		0.54	-0.21	1.29
1990	25-49 years	Deaths	Actual	0.90			
1995	25-49 years	Deaths	Actual	0.95			
2000	25-49 years	Deaths	Actual	0.90			
2005	25-49 years	Deaths	Actual	0.77			
2010	25-49 years	Deaths	Actual	0.72			
2015	25-49 years	Deaths	Actual	0.68			
2020	25-49 years	Deaths	Actual	0.64			
2025	25-49 years	Deaths	Forecast		0.63	0.56	0.69
2030	25-49 years	Deaths	Forecast		0.63	0.47	0.78

2035	25-49 years	Deaths	Forecast	0.63	0.41	0.85
2040	25-49 years	Deaths	Forecast	0.63	0.35	0.90
2045	25-49 years	Deaths	Forecast	0.63	0.30	0.95
2050	25-49 years	Deaths	Forecast	0.63	0.26	0.99
